# Supplementary material for: Targeting SLC7A11-mediated cysteine metabolism for the treatment of trastuzumab-resistant HER2-positive breast cancer
Source: eLife. 2025 Jun 4;14:RP103953. doi: 10.7554/eLife.103953 (PMC12136593; doi:10.7554/eLife.103953)
Supplement: Figure 2—source data 1. [file elife-103953-fig2-data1.zip › Figure 2-source data 1/Figure 2G.pdf]

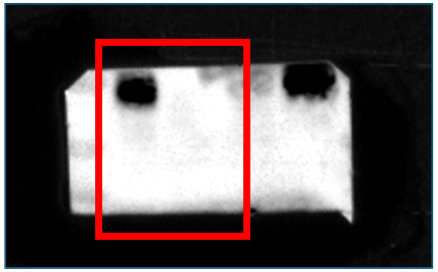

SLC7A11

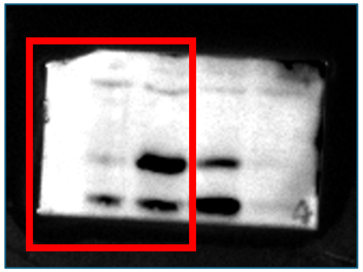

GPX4

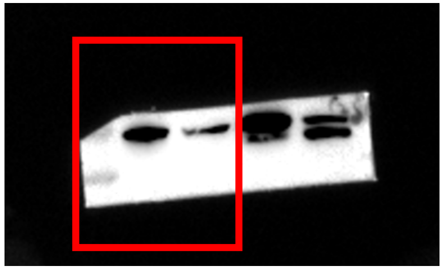

Glutathione Synthetase

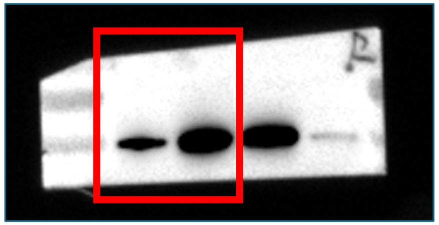

Glutathione Reductase

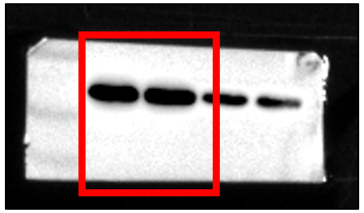

GAPDH
