# Supplementary figures and images for: Targeting SLC7A11-mediated cysteine metabolism for the treatment of trastuzumab-resistant HER2-positive breast cancer
Source: eLife. 2025 Jun 4;14:RP103953. doi: 10.7554/eLife.103953 (PMC12136593; doi:10.7554/eLife.103953)

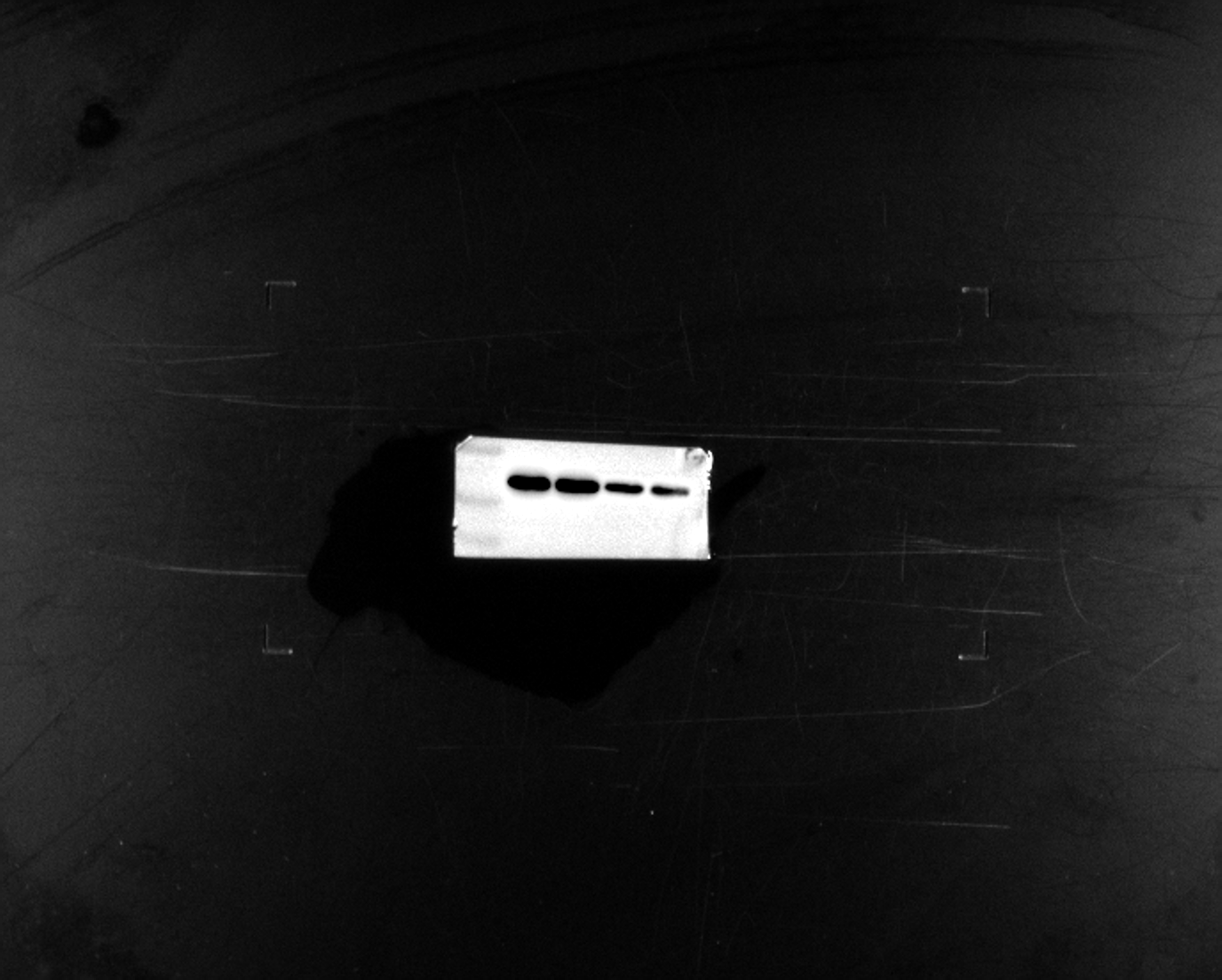

Supplement: Figure 2—source data 2. [file elife-103953-fig2-data2.zip › Figure 2-source data 2/Figure 2G-JIMT1, SKBR3-GAPDH.Tif]

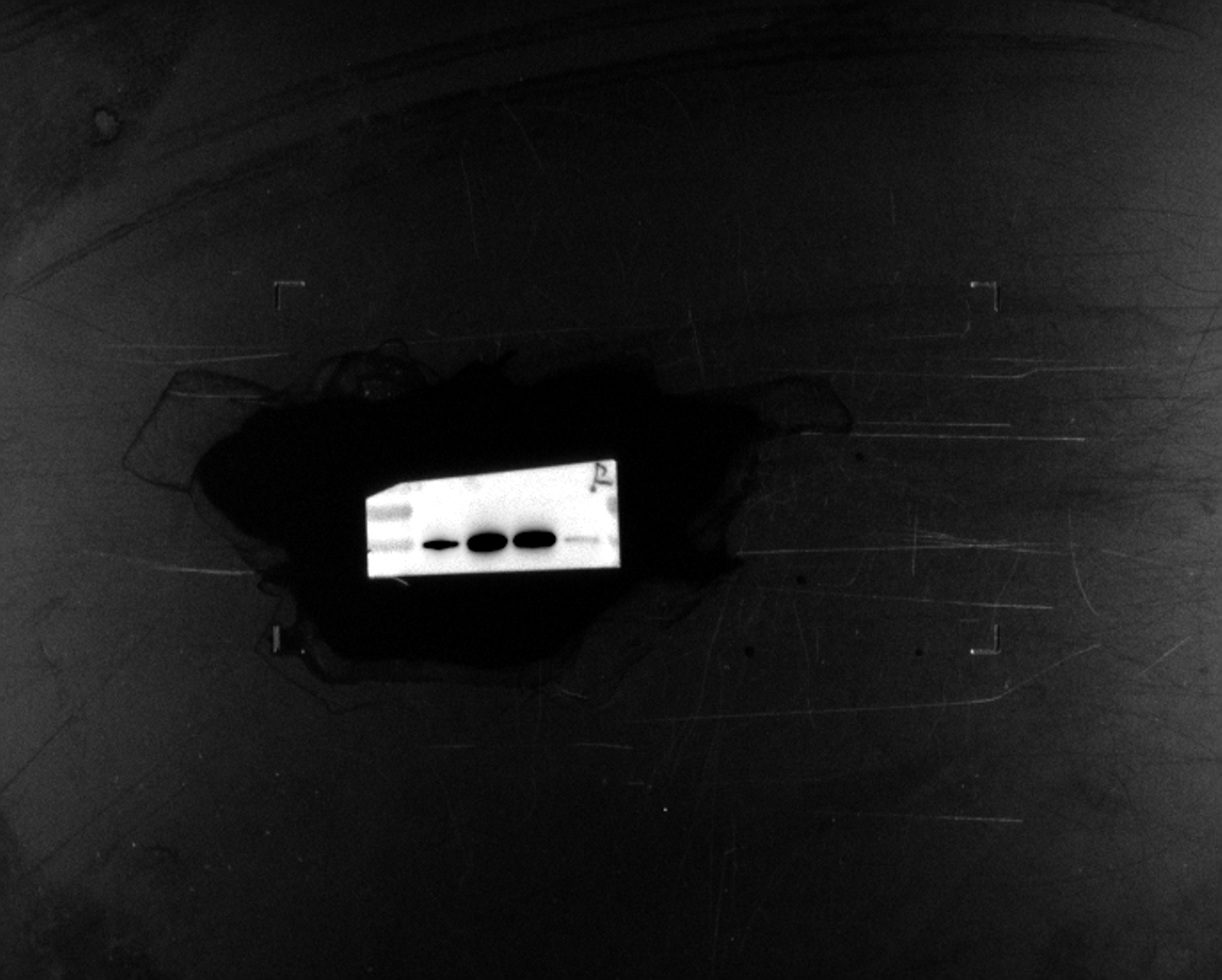

Supplement: Figure 2—source data 2. [file elife-103953-fig2-data2.zip › Figure 2-source data 2/Figure 2G-JIMT1, SKBR3-Glutathione Reductase.Tif]

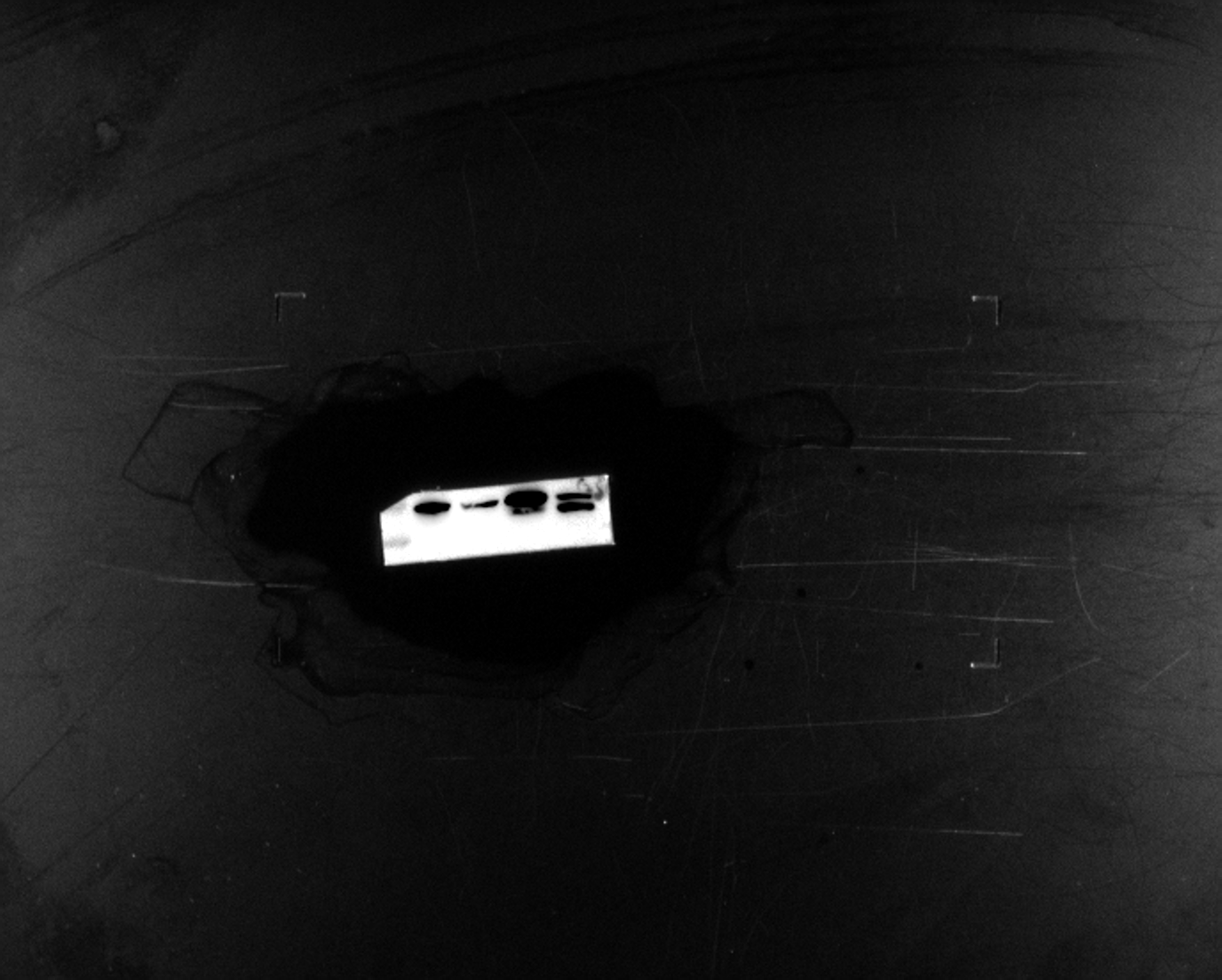

Supplement: Figure 2—source data 2. [file elife-103953-fig2-data2.zip › Figure 2-source data 2/Figure 2G-JIMT1, SKBR3-Glutathione Synthetase.Tif]

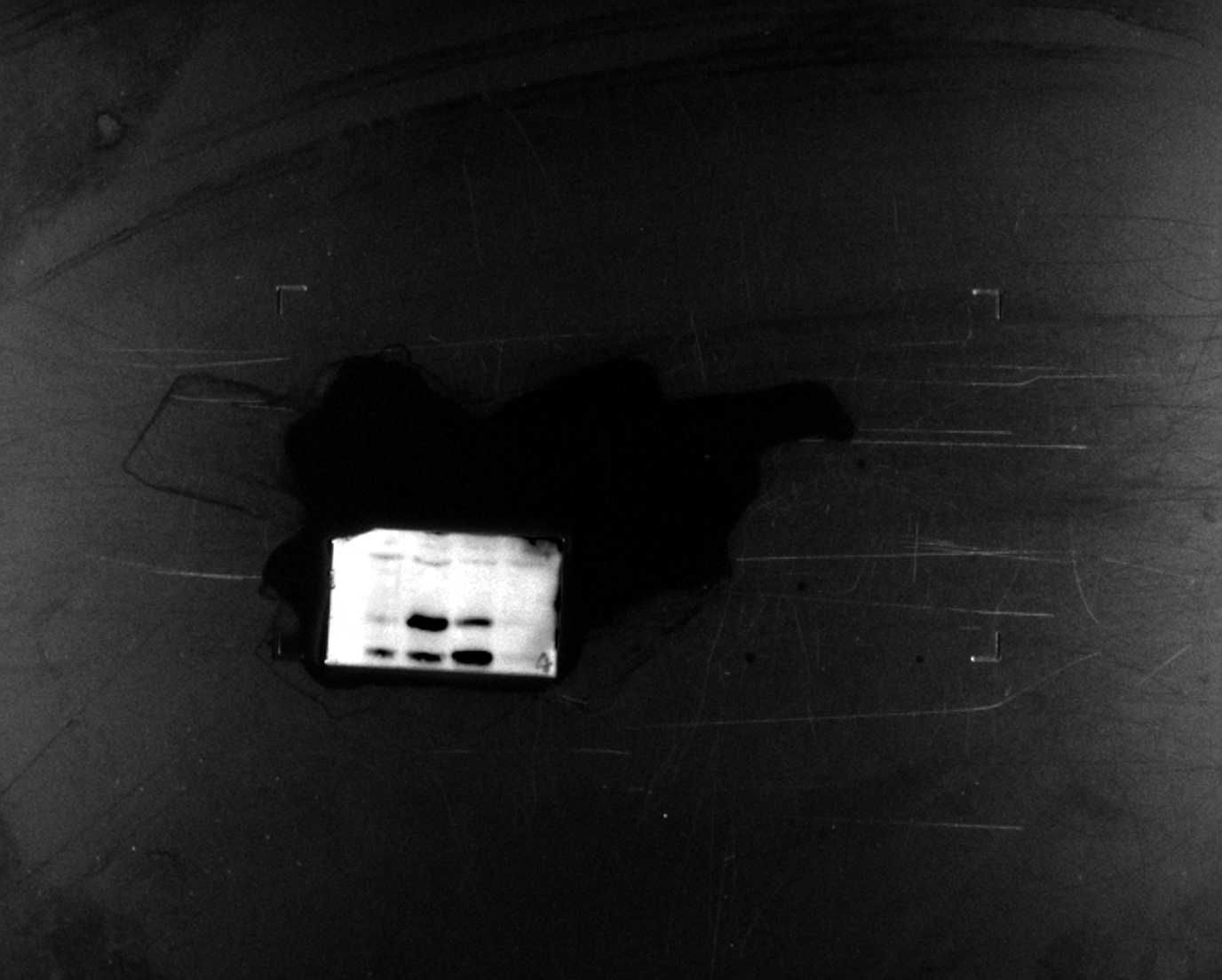

Supplement: Figure 2—source data 2. [file elife-103953-fig2-data2.zip › Figure 2-source data 2/Figure 2G-JIMT1, SKBR3-GPX4.Tif]

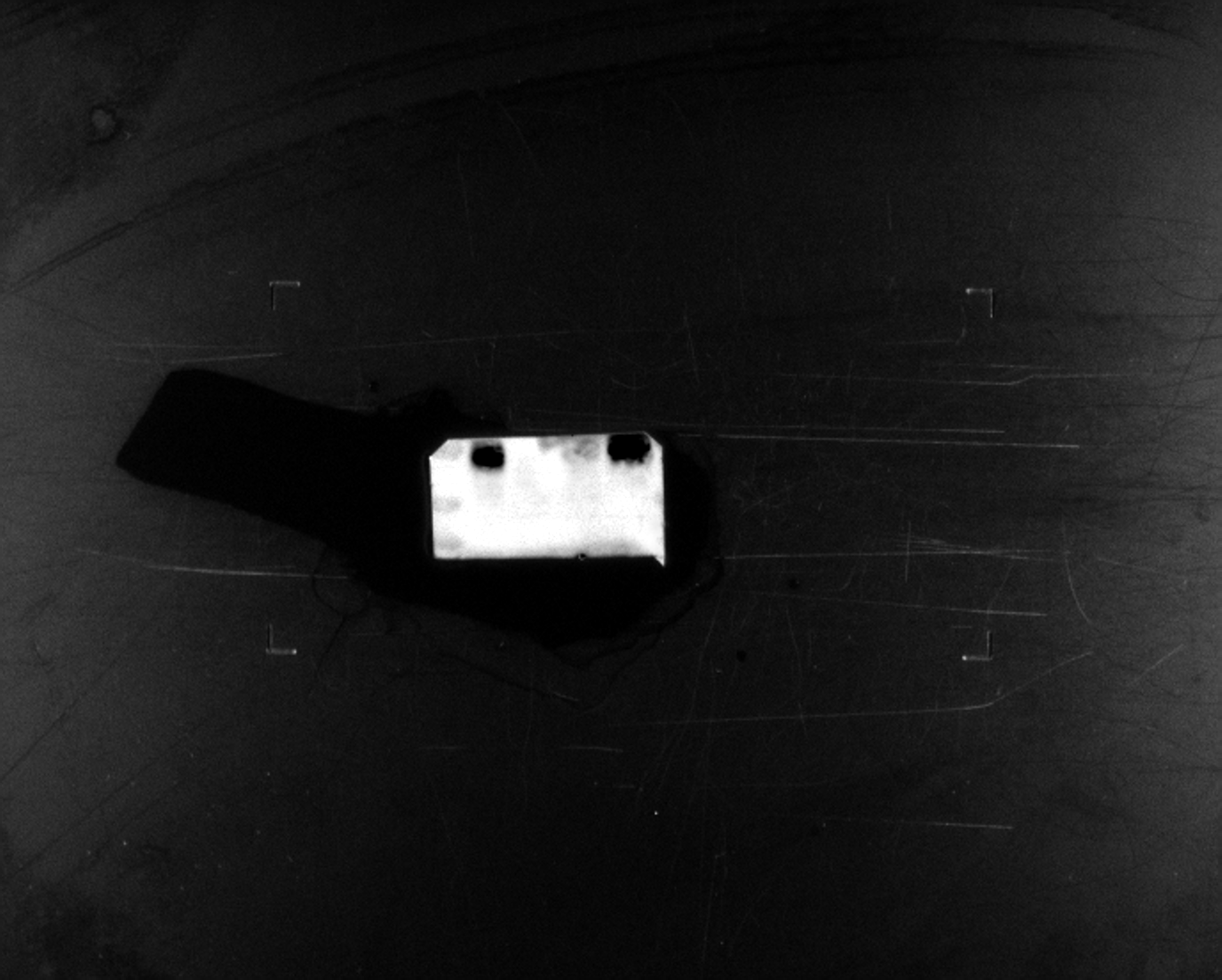

Supplement: Figure 2—source data 2. [file elife-103953-fig2-data2.zip › Figure 2-source data 2/Figure 2G-JIMT1, SKBR3-SLC7A11.Tif]

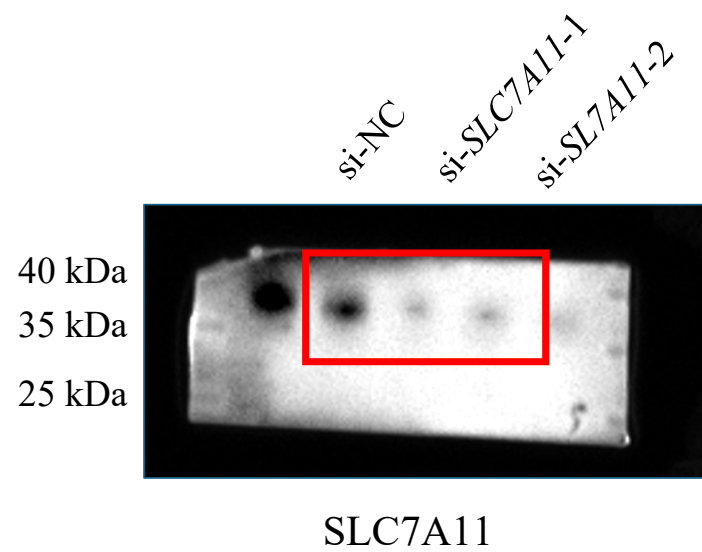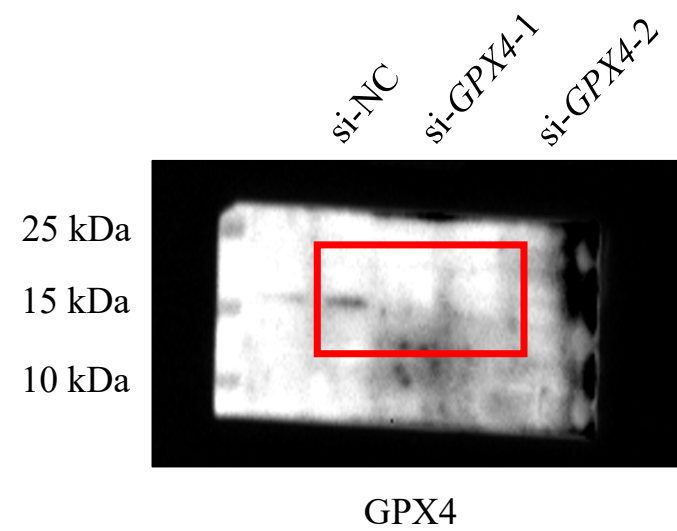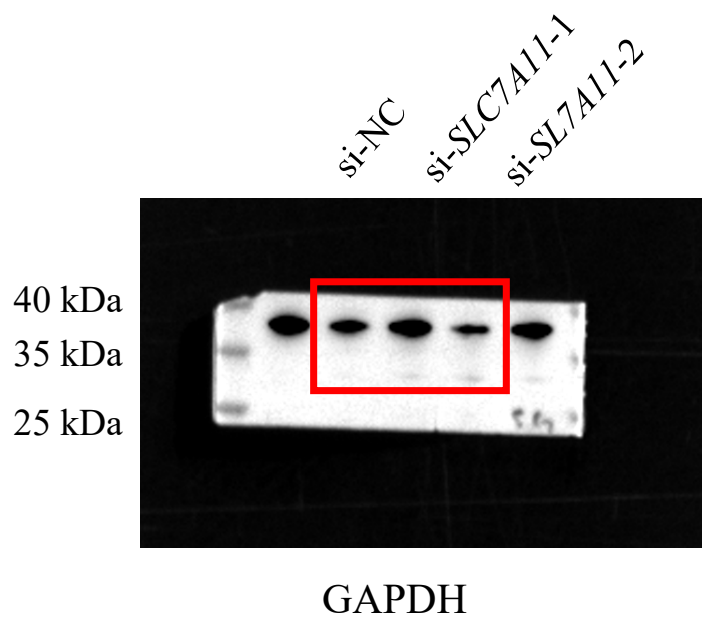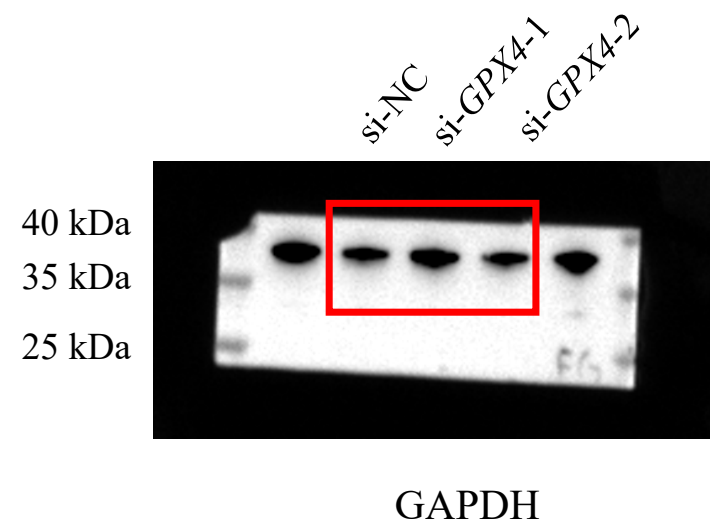

Supplement: Figure 3—source data 1. [file elife-103953-fig3-data1.zip › Figure 3-source data 1/Figure 3A,B.pdf]

Trastuzumab

0h 24h 48h 72h

40 kDa

35 kDa

25 kDa

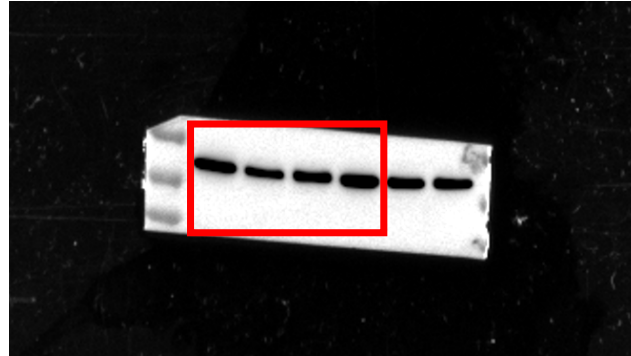

SLC7A11

40 kDa

35 kDa

25 kDa

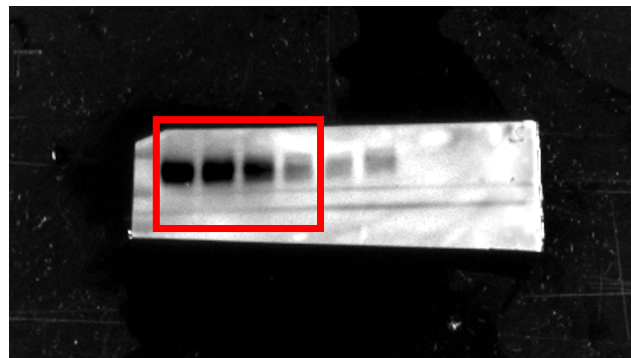

GAPDH

Supplement: Figure 3—source data 1. [file elife-103953-fig3-data1.zip › Figure 3-source data 1/Figure 3I.pdf]

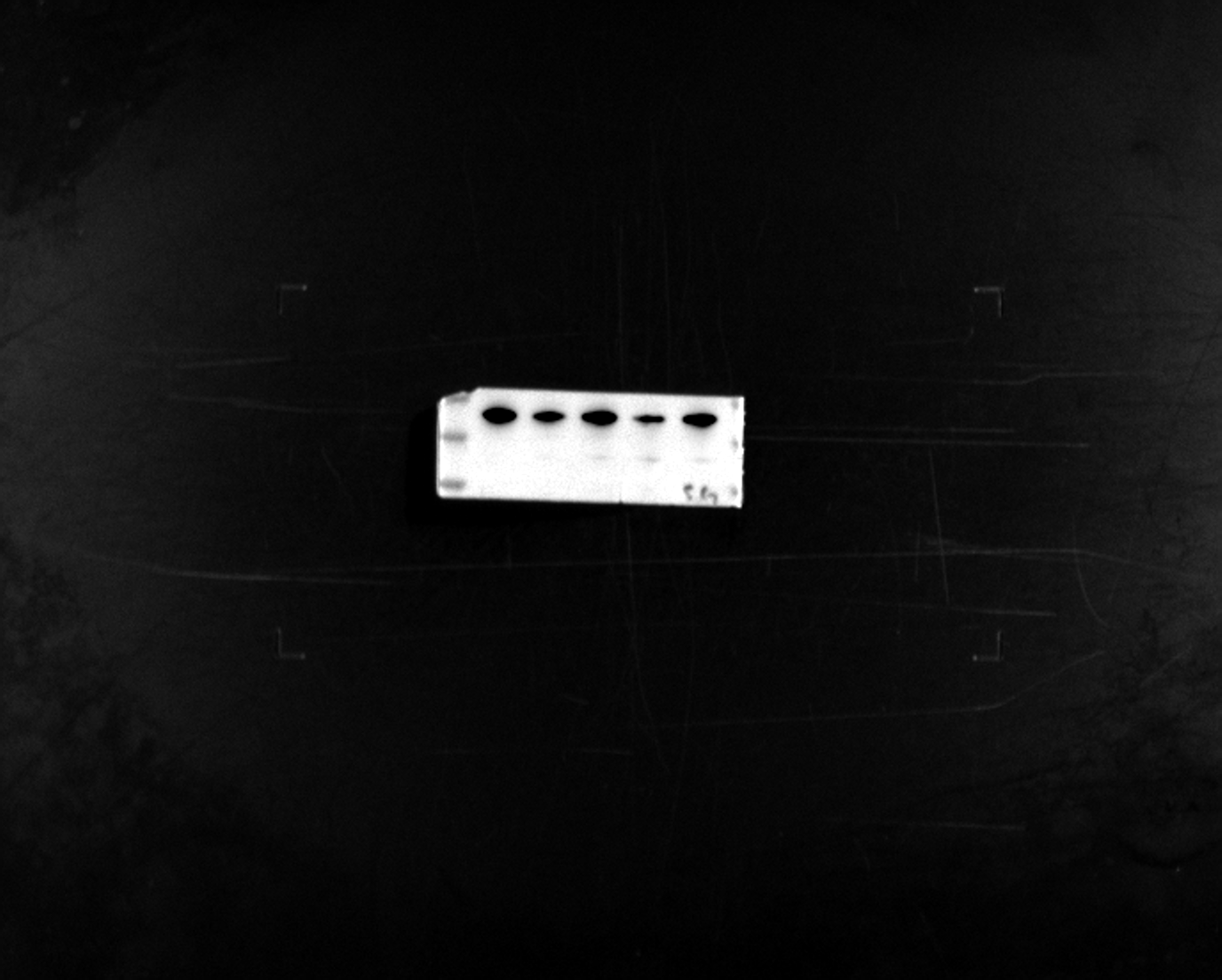

Supplement: Figure 3—source data 2. [file elife-103953-fig3-data2.zip › Figure 3-source data 2/Figure 3A-JIMT1-siSLC7A11-GAPDH.Tif]

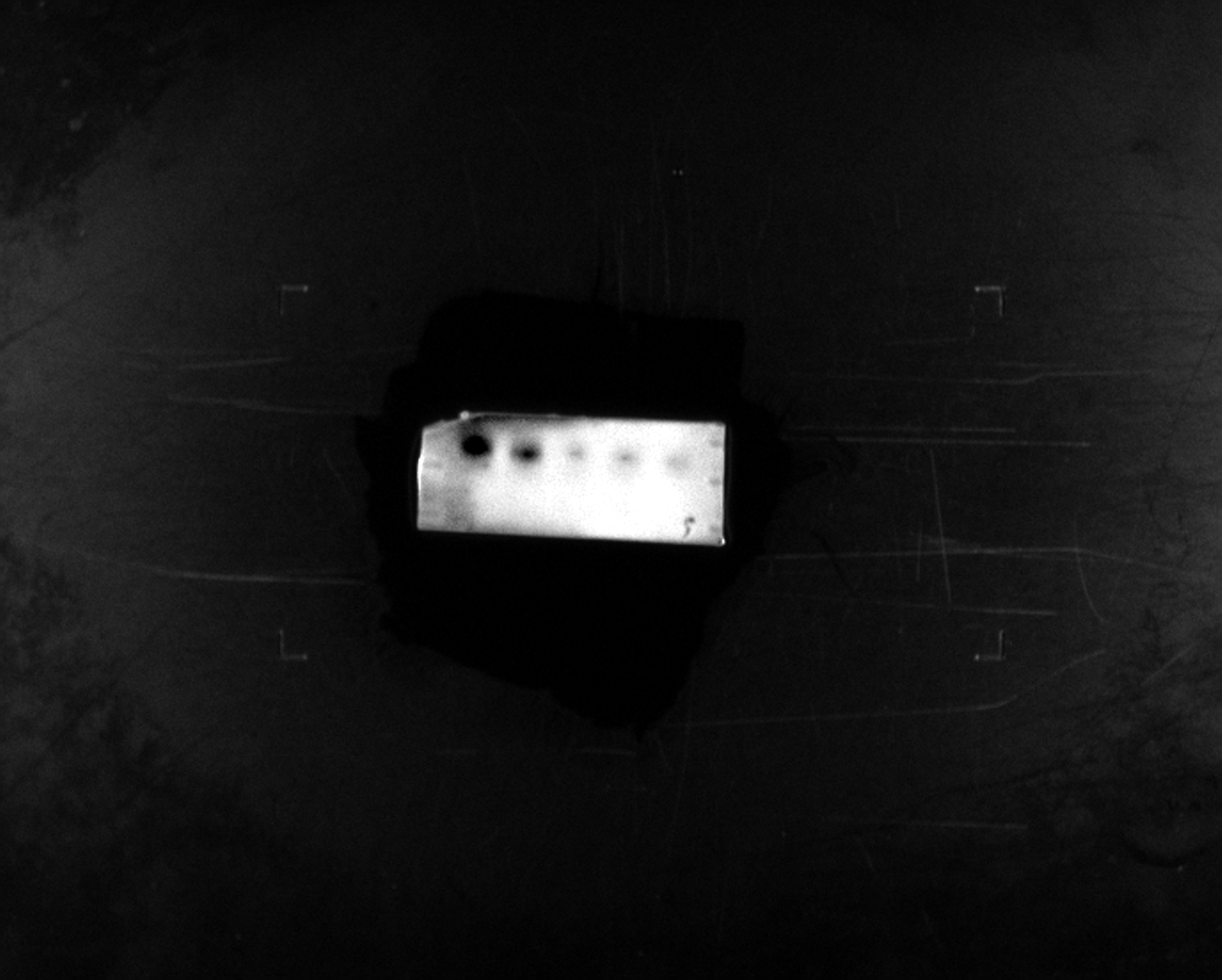

Supplement: Figure 3—source data 2. [file elife-103953-fig3-data2.zip › Figure 3-source data 2/Figure 3A-JIMT1-siSLC7A11-SLC7A11.Tif]

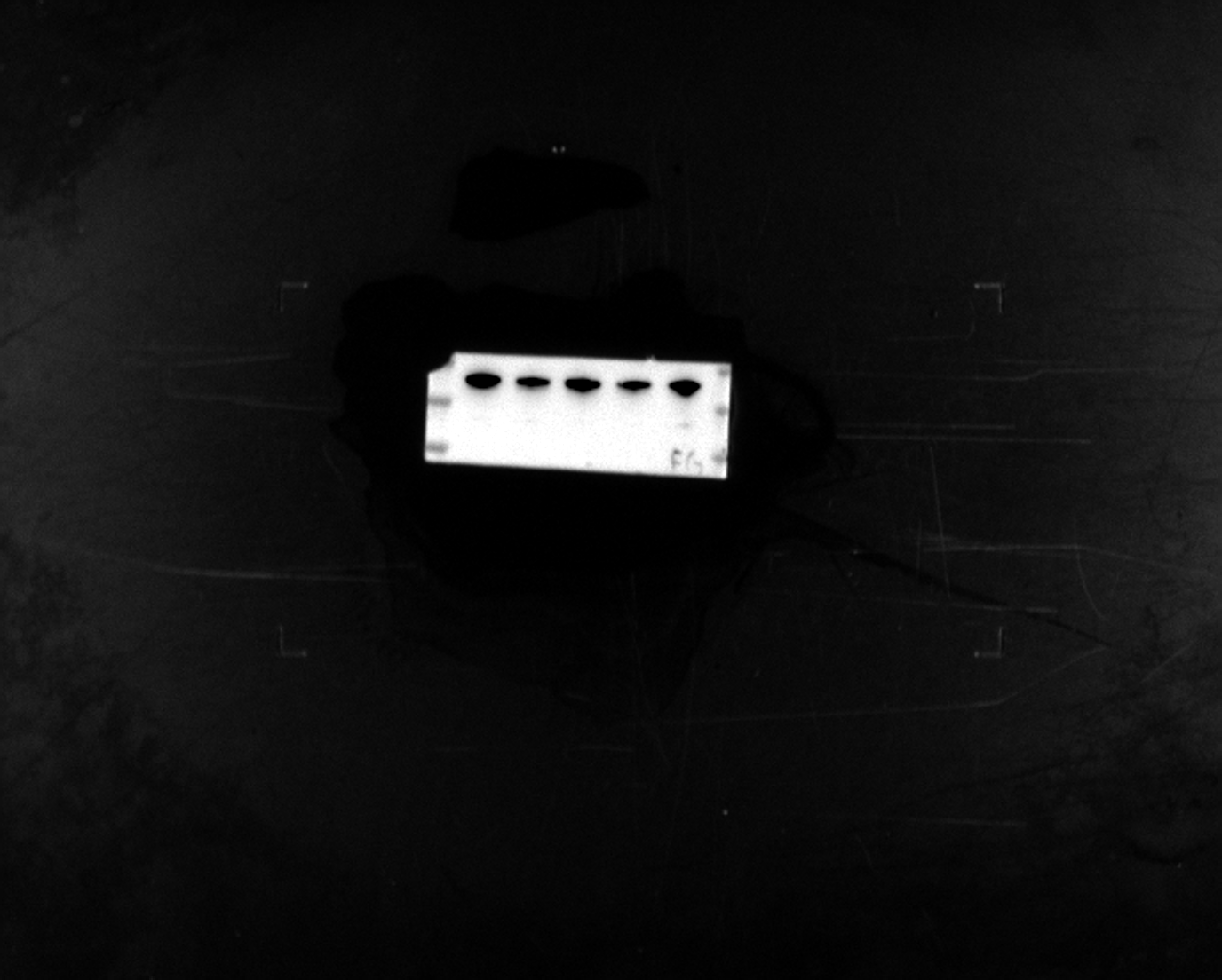

Supplement: Figure 3—source data 2. [file elife-103953-fig3-data2.zip › Figure 3-source data 2/Figure 3B-JIMT1-siGPX4-GAPDH.Tif]

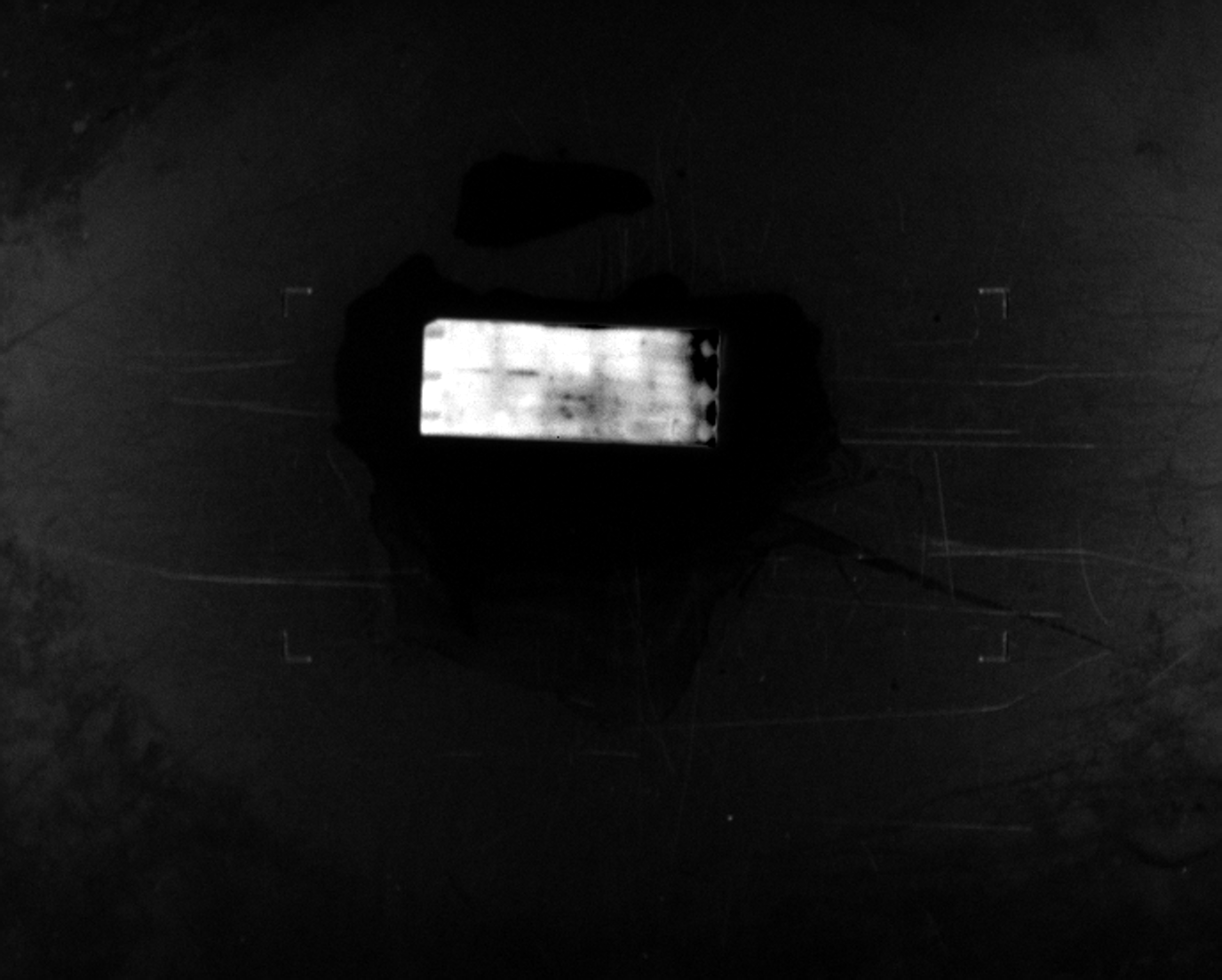

Supplement: Figure 3—source data 2. [file elife-103953-fig3-data2.zip › Figure 3-source data 2/Figure 3B-JIMT1-siGPX4-GPX4.Tif]

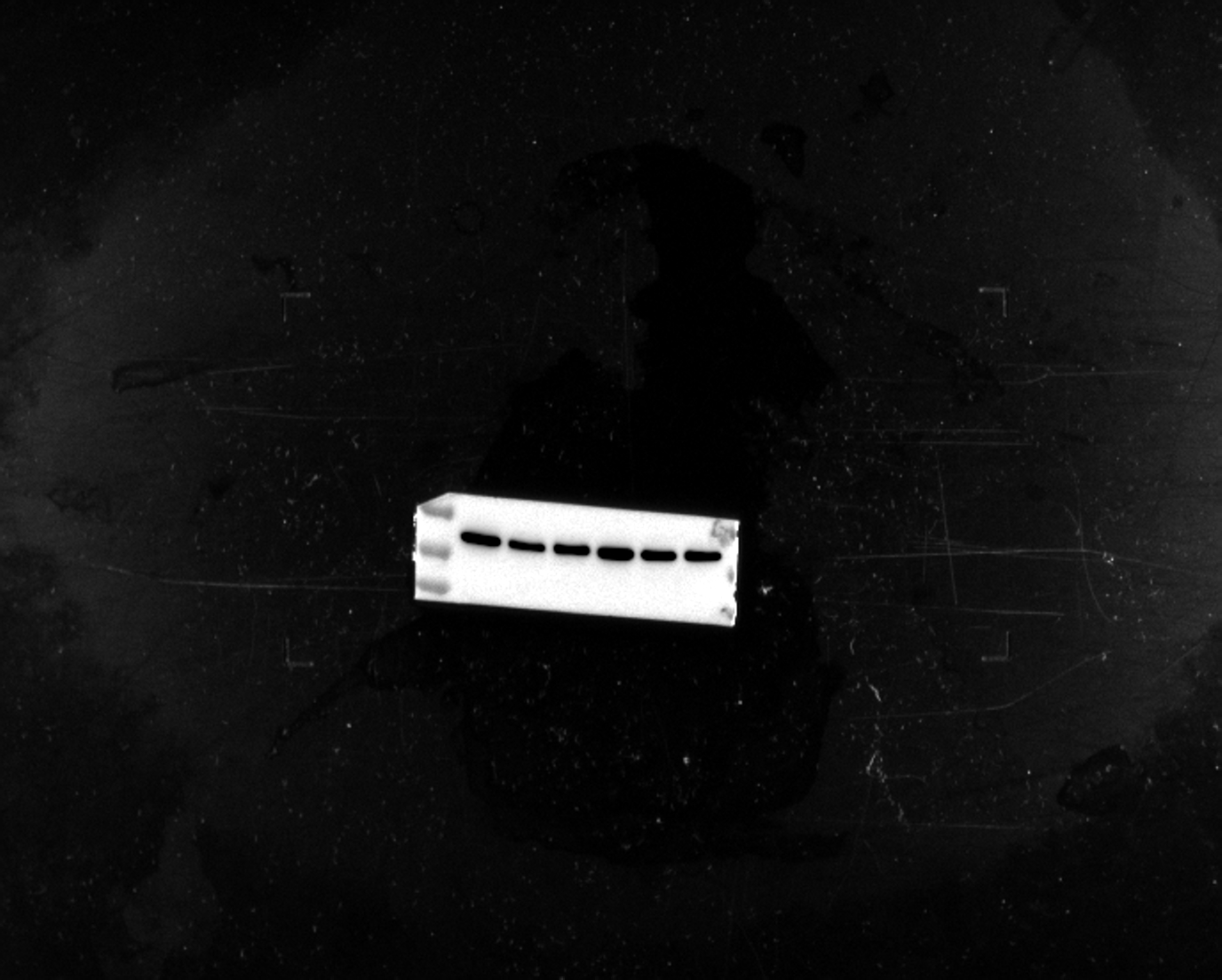

Supplement: Figure 3—source data 2. [file elife-103953-fig3-data2.zip › Figure 3-source data 2/Figure 3I-JIMT1-GAPDH.Tif]

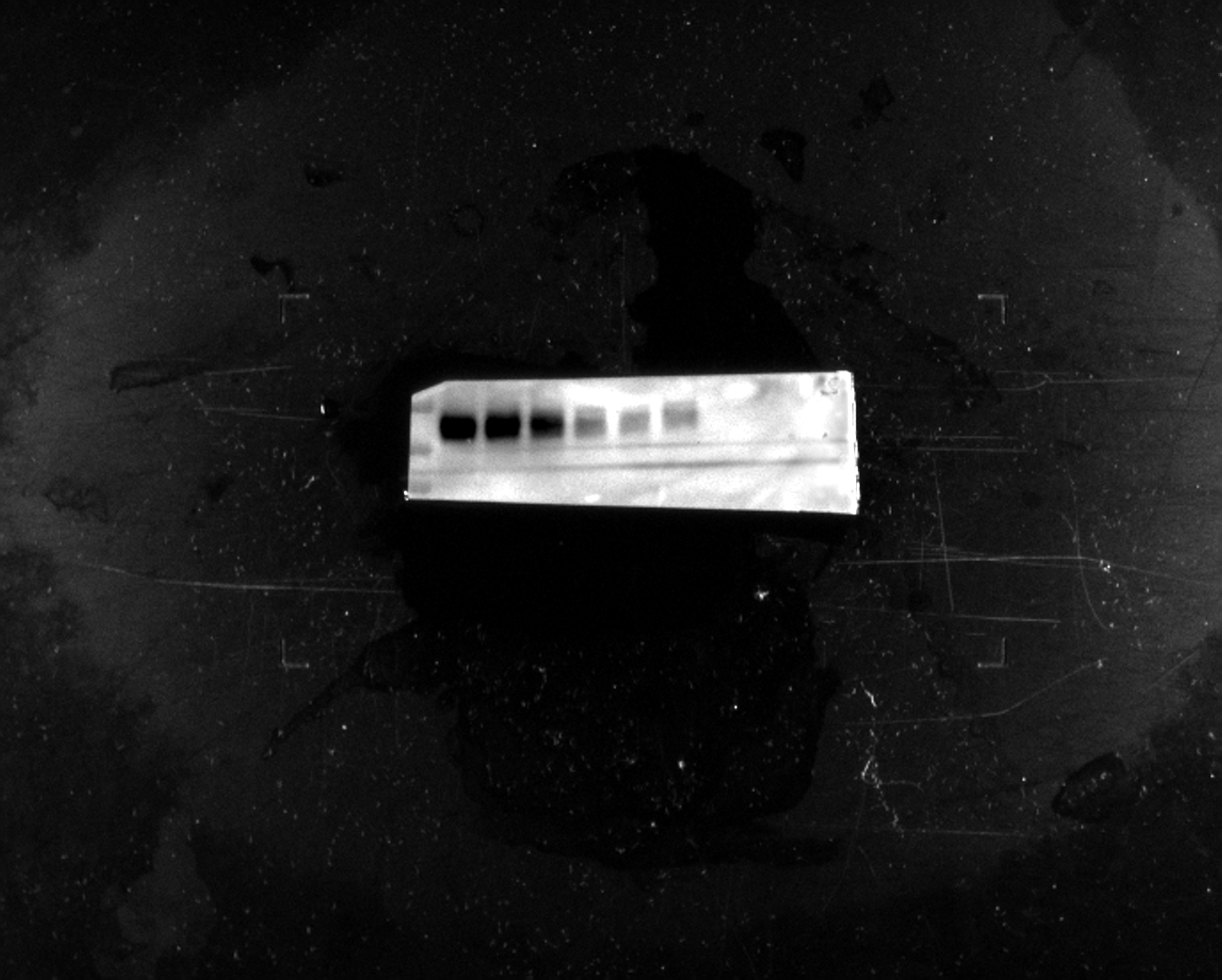

Supplement: Figure 3—source data 2. [file elife-103953-fig3-data2.zip › Figure 3-source data 2/Figure 3I-JIMT1-SLC7A11.Tif]

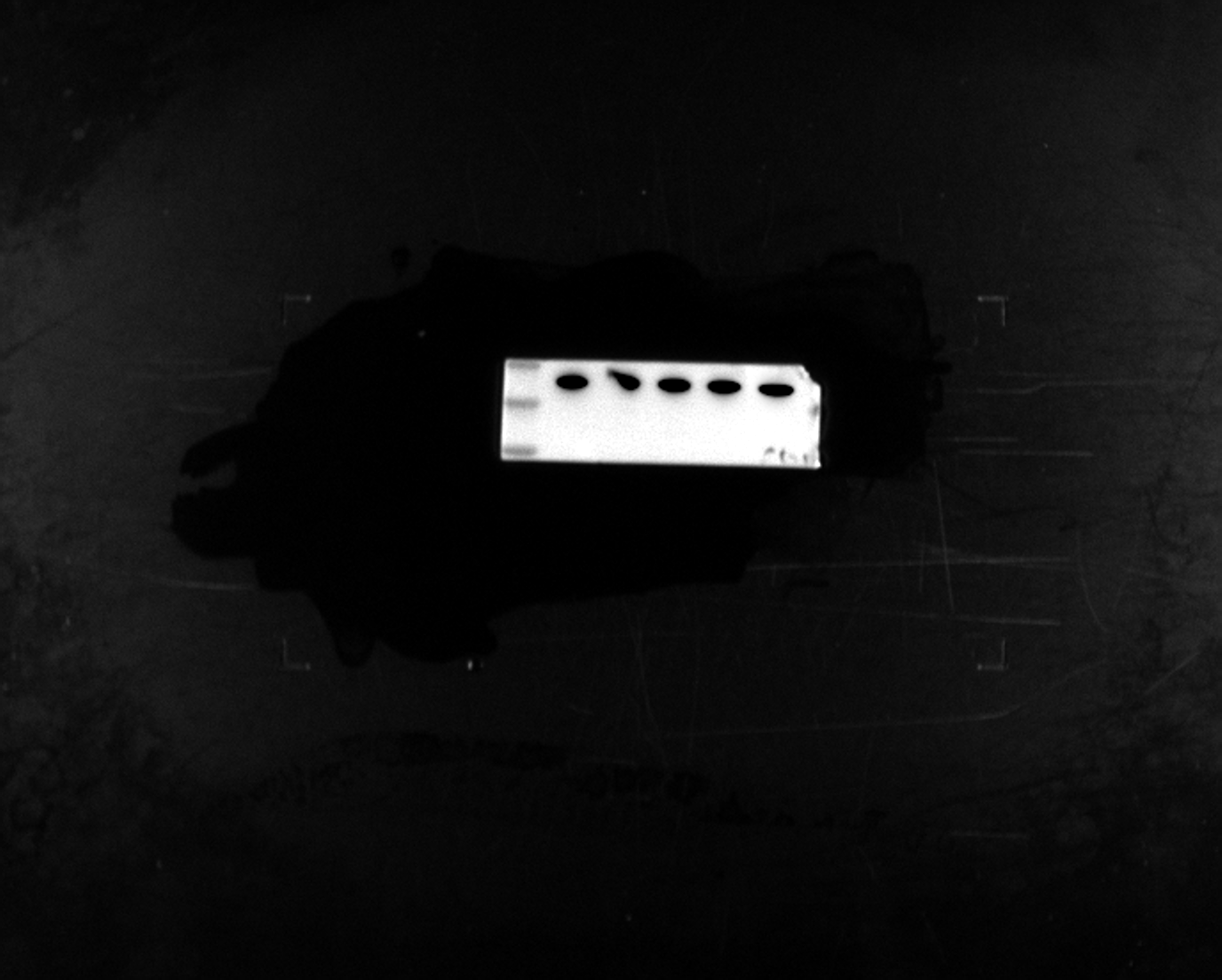

Supplement: Figure 3—figure supplement 1—source data 2. [file elife-103953-fig3-figsupp1-data2.zip › Figure 3-figure supplement 1-source data 2/Figure 3-figure supplement 1A-SKBR3-siSLC7A11-GAPDH.Tif]

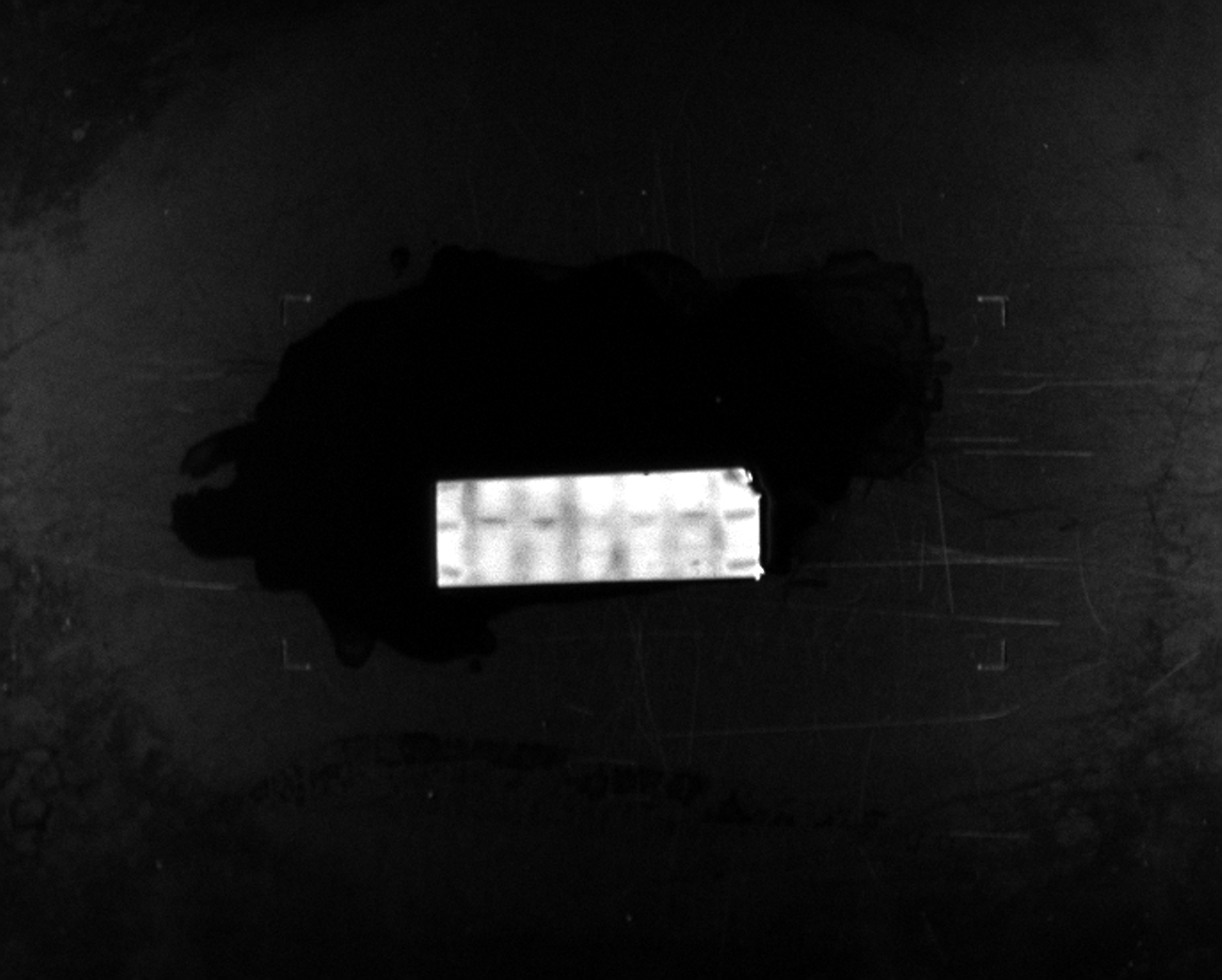

Supplement: Figure 3—figure supplement 1—source data 2. [file elife-103953-fig3-figsupp1-data2.zip › Figure 3-figure supplement 1-source data 2/Figure 3-figure supplement 1A-SKBR3-siSLC7A11-SLC7A11.Tif]

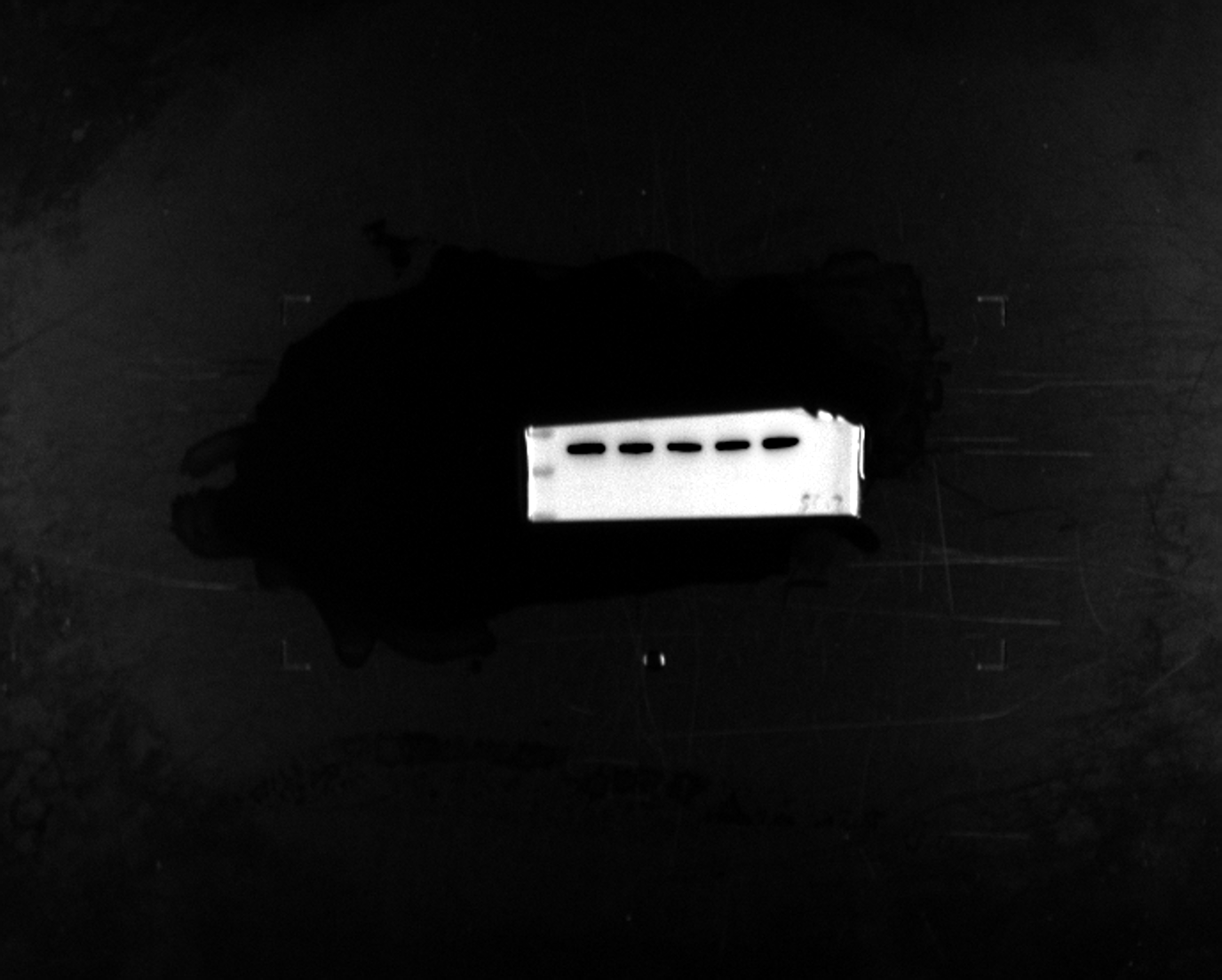

Supplement: Figure 3—figure supplement 1—source data 2. [file elife-103953-fig3-figsupp1-data2.zip › Figure 3-figure supplement 1-source data 2/Figure 3-figure supplement 1B-SKBR3-siGPX4-GAPDH.Tif]

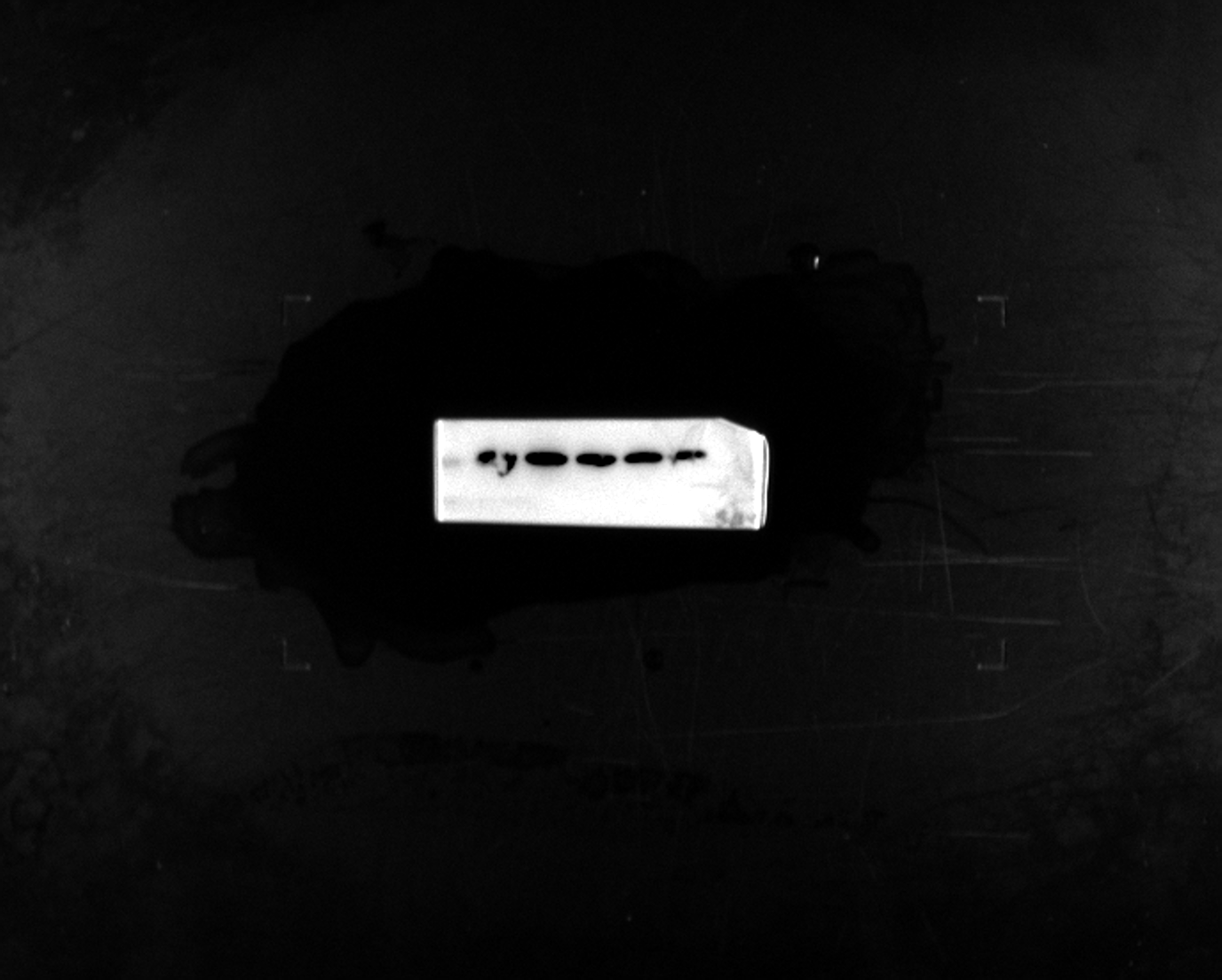

Supplement: Figure 3—figure supplement 1—source data 2. [file elife-103953-fig3-figsupp1-data2.zip › Figure 3-figure supplement 1-source data 2/Figure 3-figure supplement 1B-SKBR3-siGPX4-GPX4.Tif]

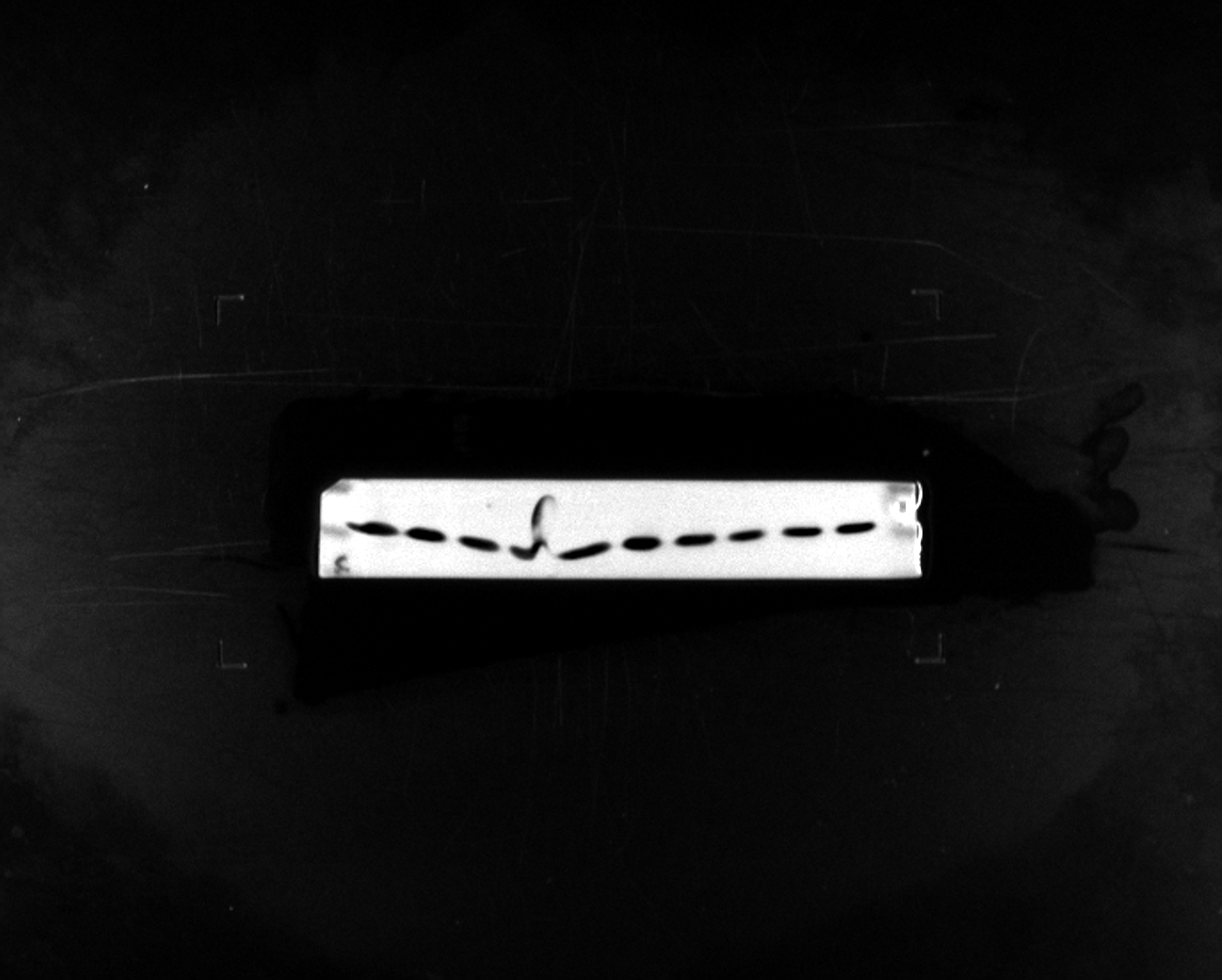

Supplement: Figure 5—source data 2. [file elife-103953-fig5-data2.zip › Figure 5-source data 2/Figure 5D-JIMT1-H3.tif]

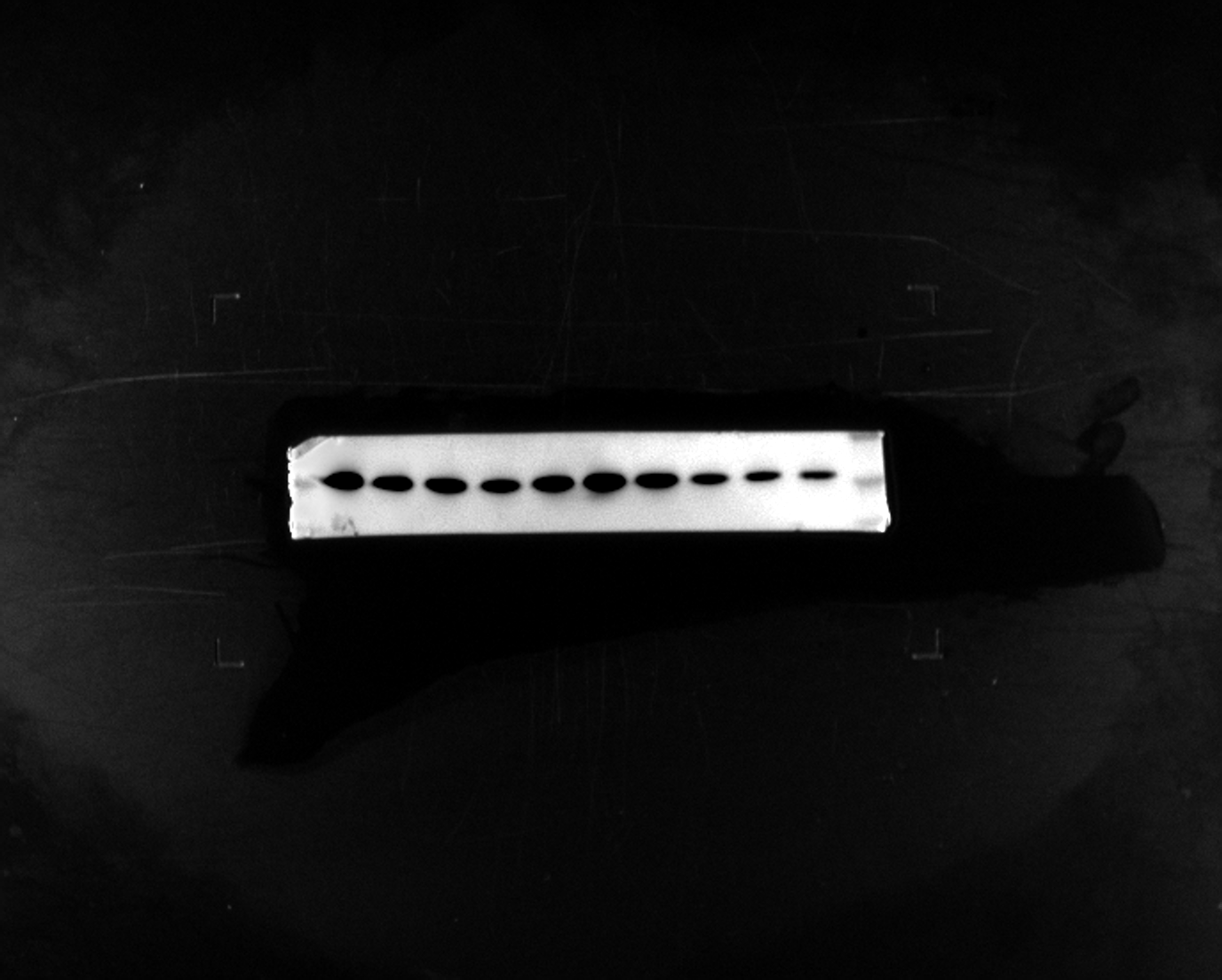

Supplement: Figure 5—source data 2. [file elife-103953-fig5-data2.zip › Figure 5-source data 2/Figure 5D-JIMT1-H3K4me3.tif]

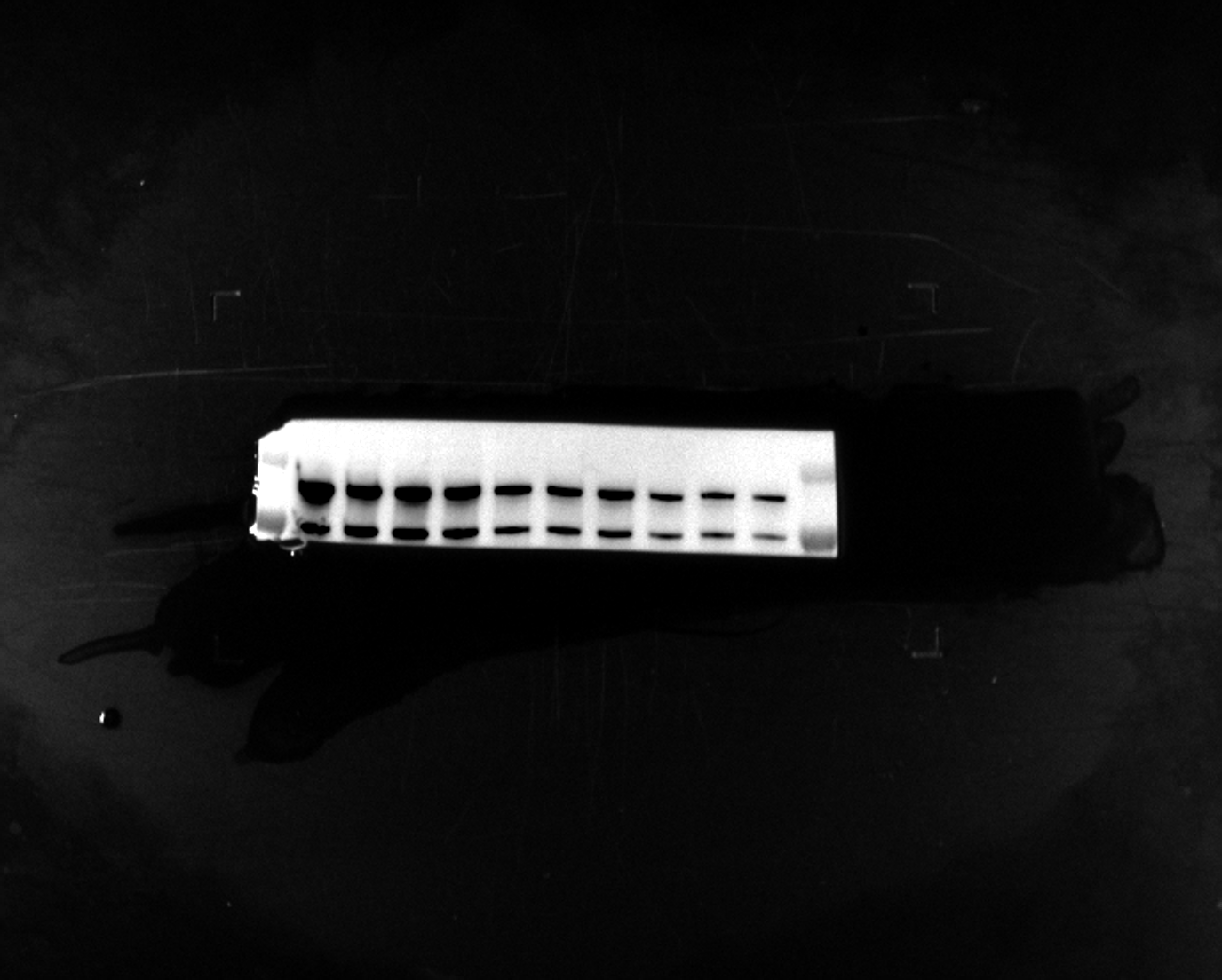

Supplement: Figure 5—source data 2. [file elife-103953-fig5-data2.zip › Figure 5-source data 2/Figure 5E-JIMT1-ASH2L.tif]

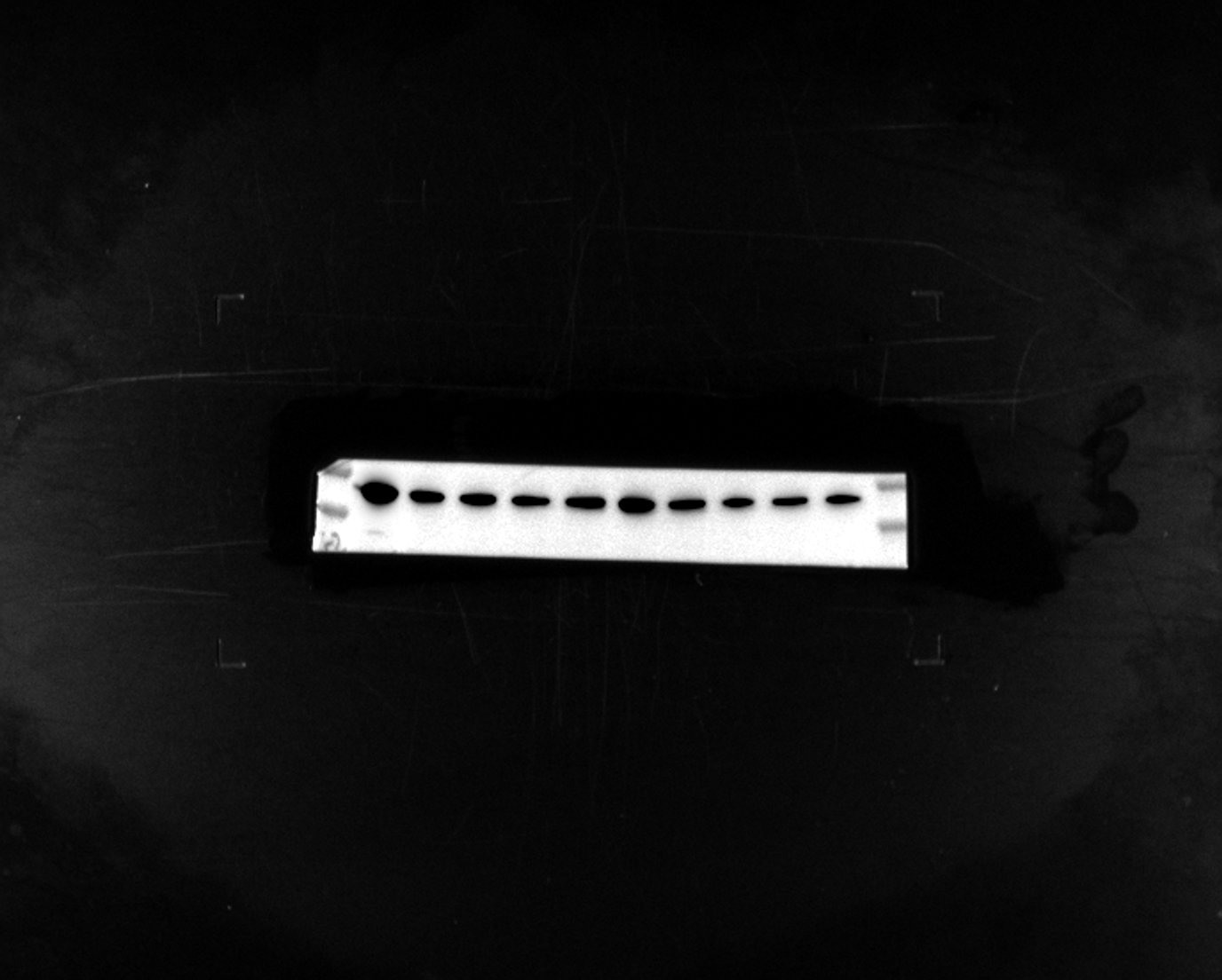

Supplement: Figure 5—source data 2. [file elife-103953-fig5-data2.zip › Figure 5-source data 2/Figure 5E-JIMT1-GAPDH.tif]

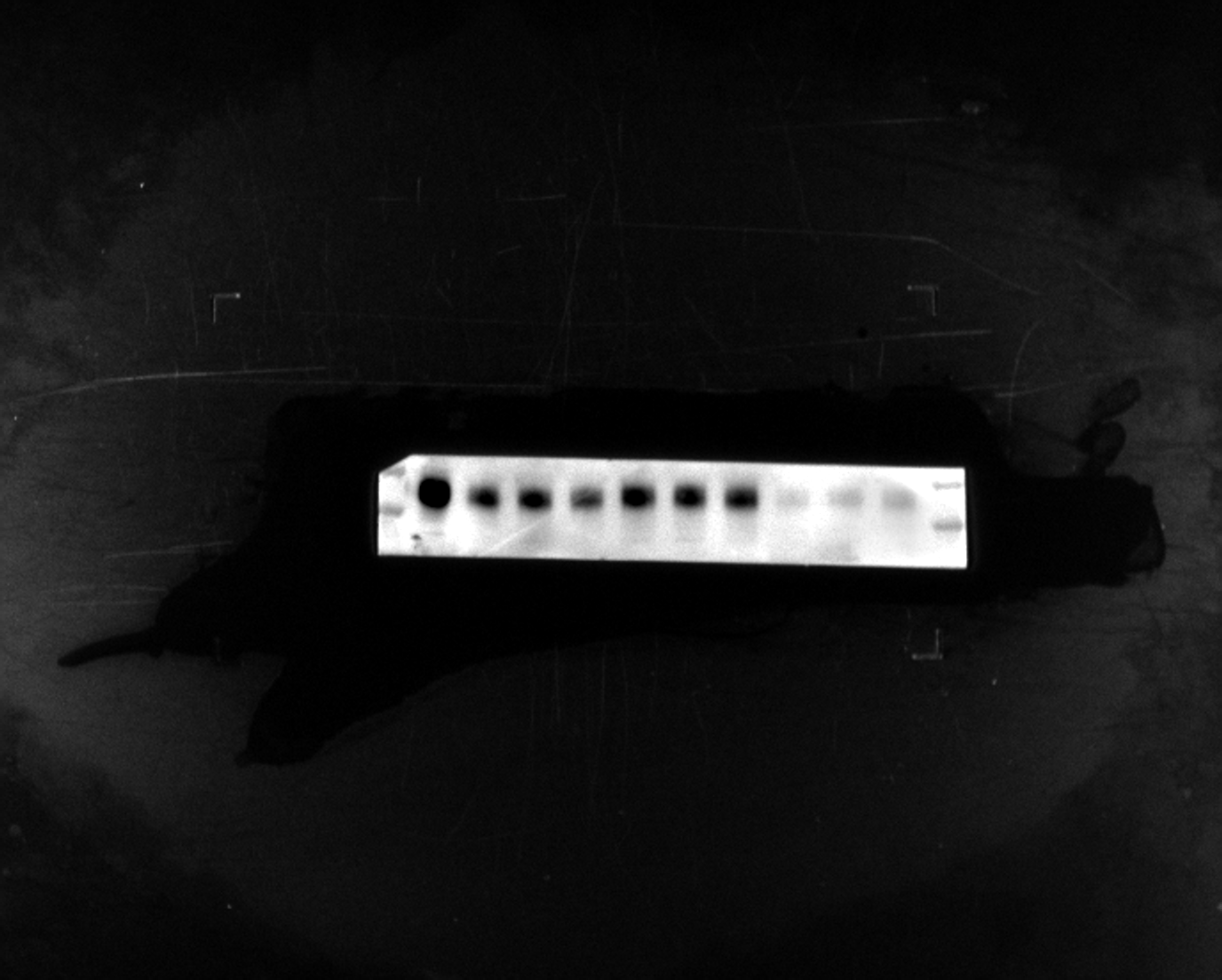

Supplement: Figure 5—source data 2. [file elife-103953-fig5-data2.zip › Figure 5-source data 2/Figure 5E-JIMT1-SLC7A11.tif]

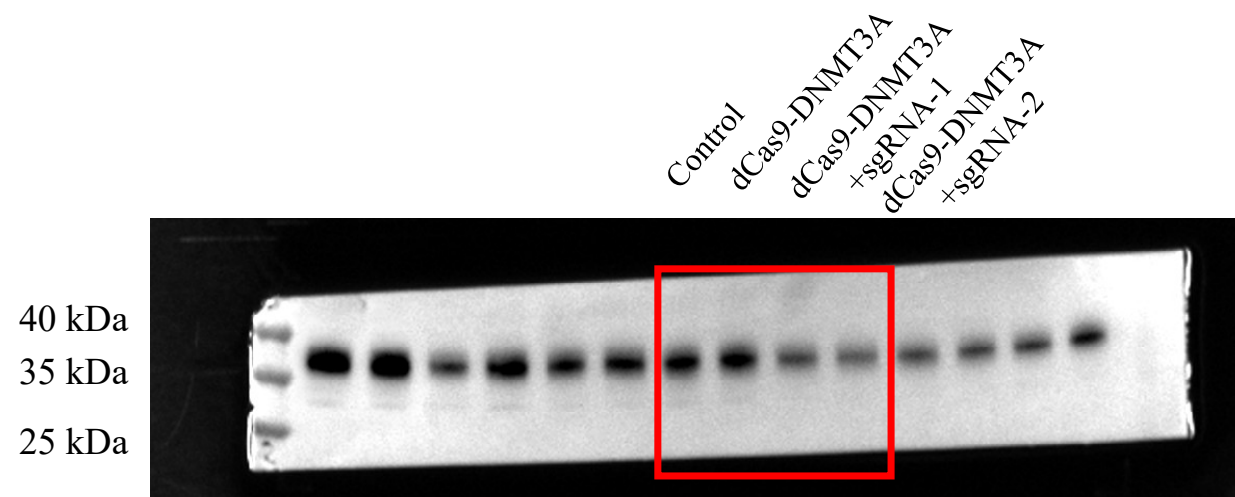

SLC7A11

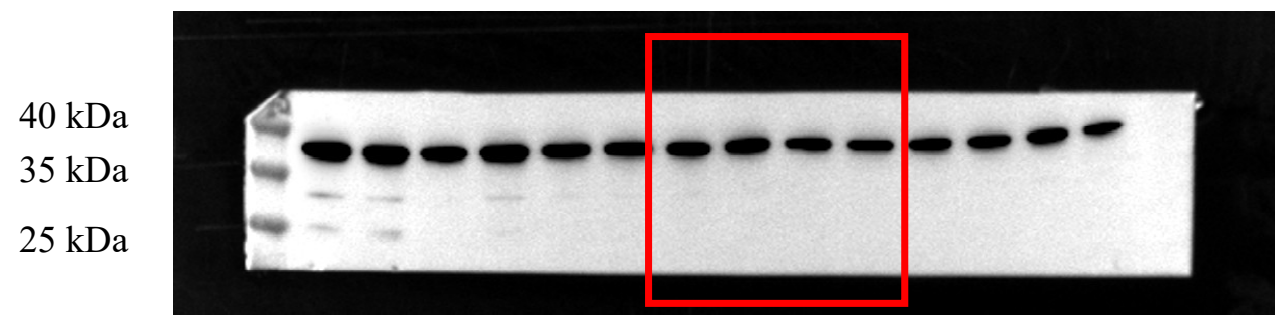

GAPDH

Supplement: Figure 6—source data 1. [file elife-103953-fig6-data1.zip › Figure 6-source data 1/Figure 6E.pdf]

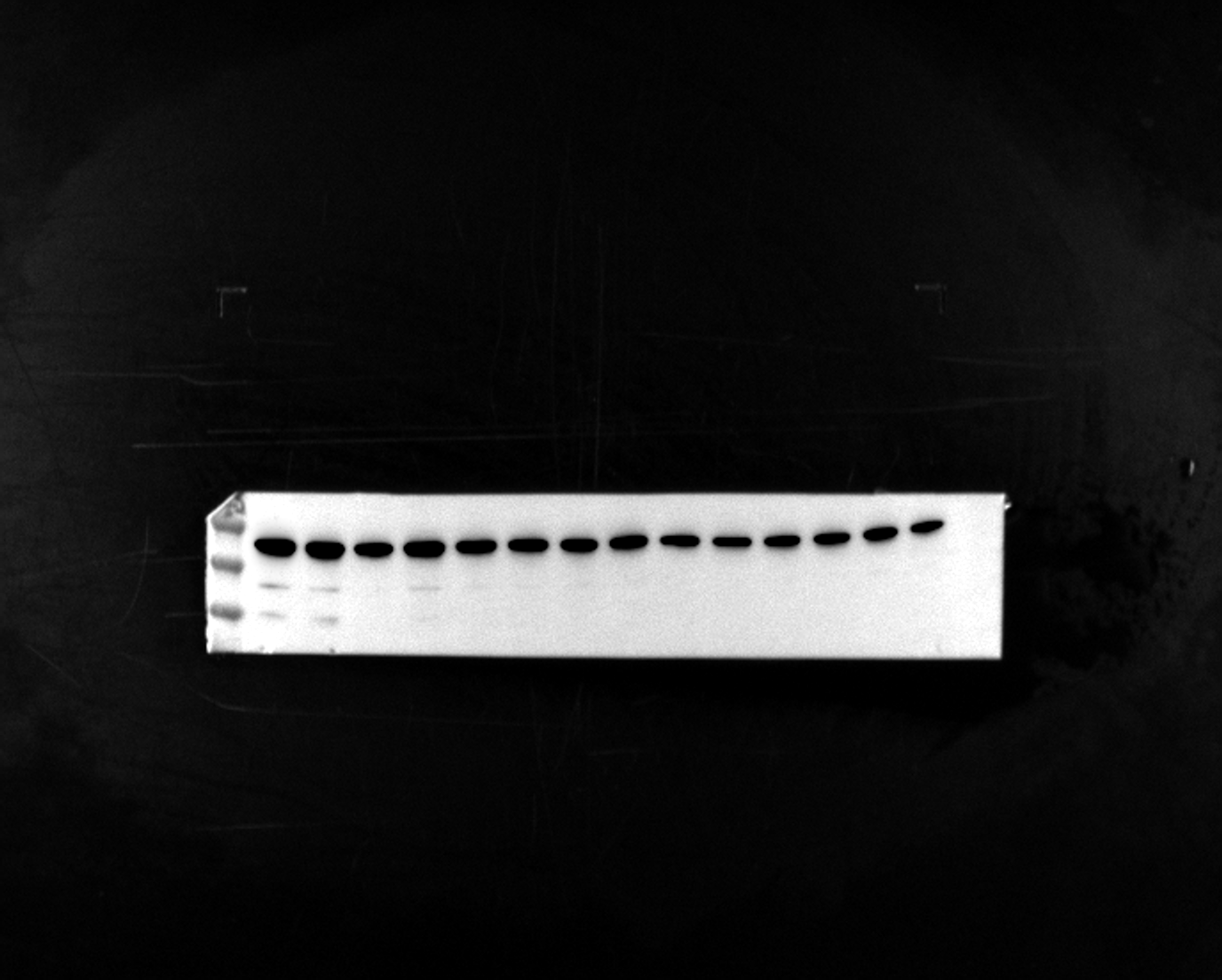

Supplement: Figure 6—source data 2. [file elife-103953-fig6-data2.zip › Figure 6-source data 2/Figure 6E-GAPDH.tif]

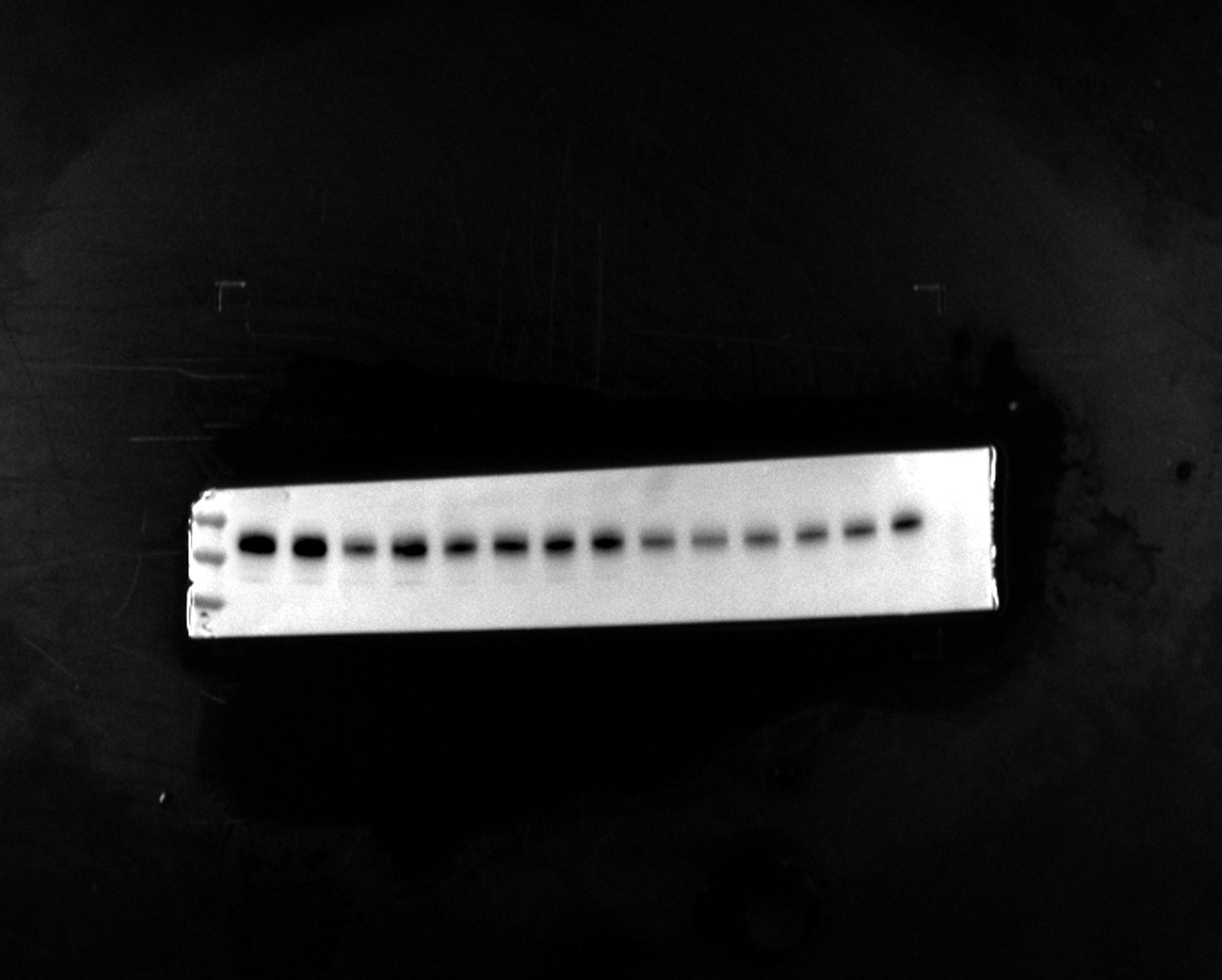

Supplement: Figure 6—source data 2. [file elife-103953-fig6-data2.zip › Figure 6-source data 2/Figure 6E-SLC7A11.tif]

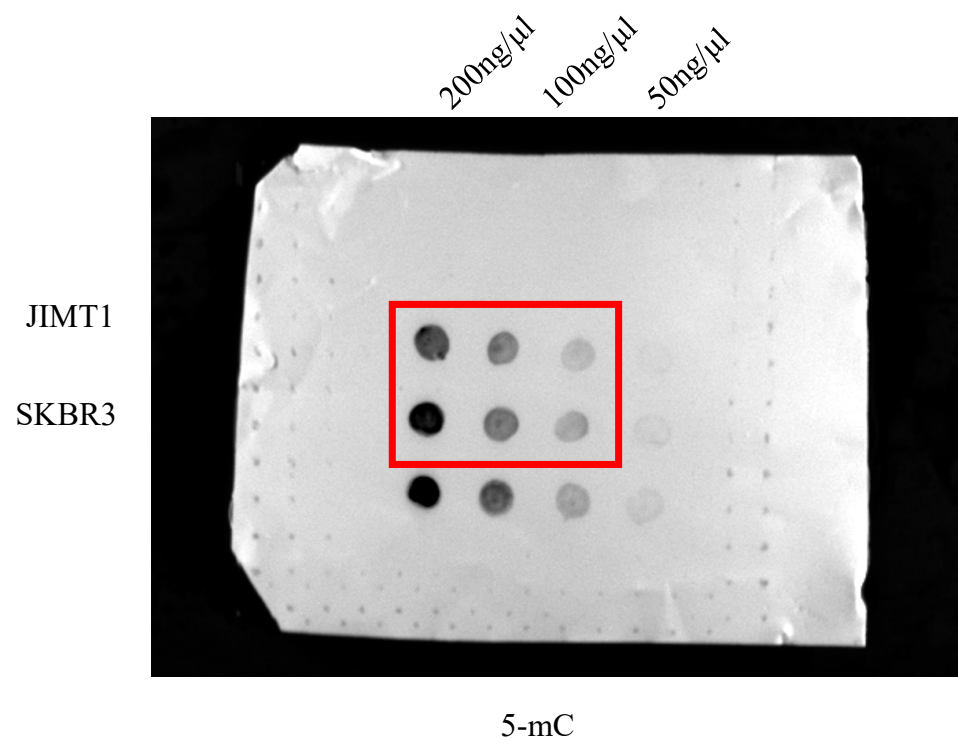

Supplement: Figure 6—figure supplement 1—source data 1. [file elife-103953-fig6-figsupp1-data1.zip › Figure 6-figure supplement 1-source data 1/Figure 6-figure supplement 1A.pdf]

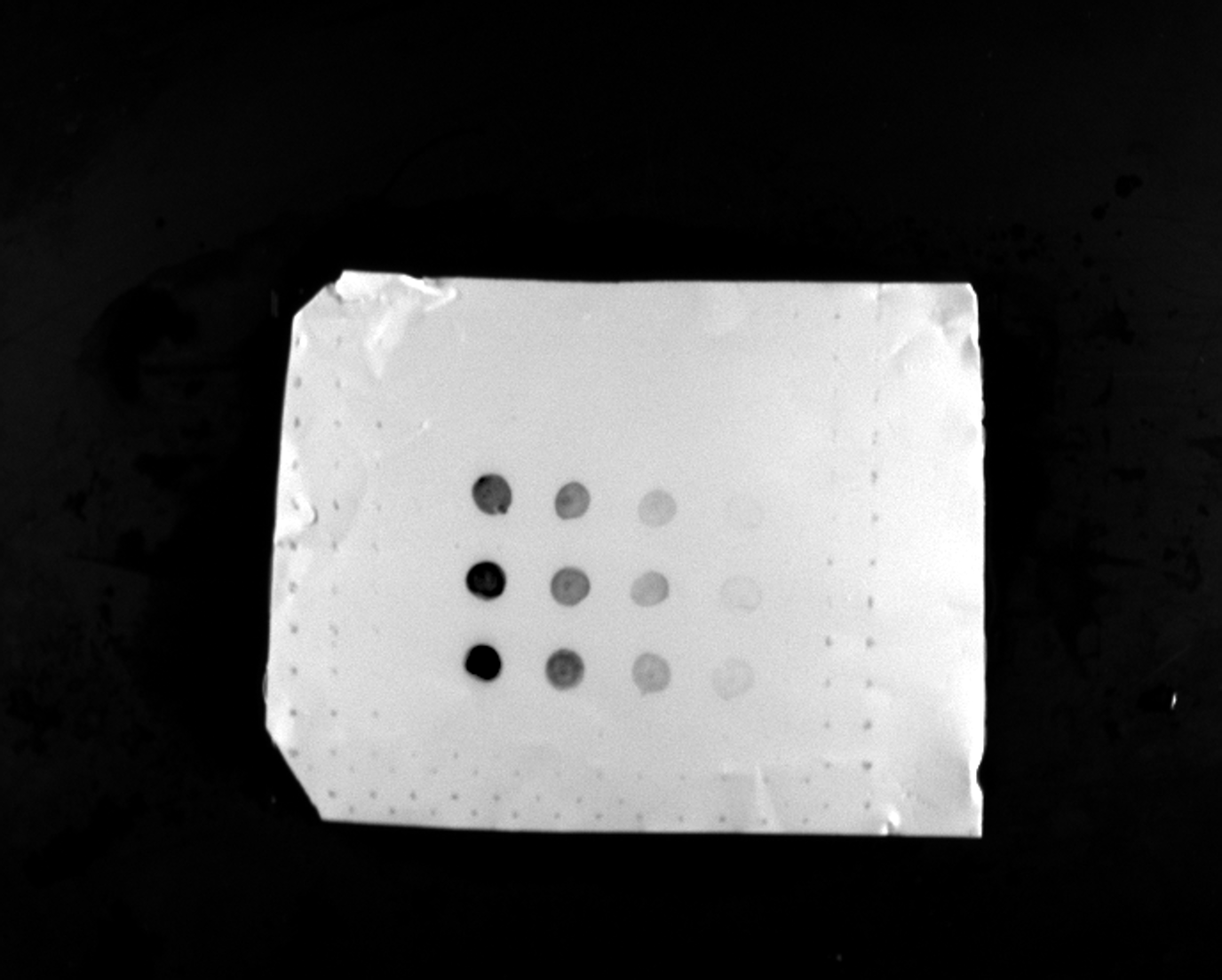

Supplement: Figure 6—figure supplement 1—source data 2. [file elife-103953-fig6-figsupp1-data2.zip › Figure 6-figure supplement 1-source data 2/Figure 6-figure supplement 1A.Tif]
