# Supplementary material for: Targeting SLC7A11-mediated cysteine metabolism for the treatment of trastuzumab-resistant HER2-positive breast cancer
Source: eLife. 2025 Jun 4;14:RP103953. doi: 10.7554/eLife.103953 (PMC12136593; doi:10.7554/eLife.103953)
Supplement: Figure 3—figure supplement 1—source data 1. [file elife-103953-fig3-figsupp1-data1.zip › Figure 3-figure supplement 1-source data 1/Figure 3-figure supplement 1A, B.pdf]

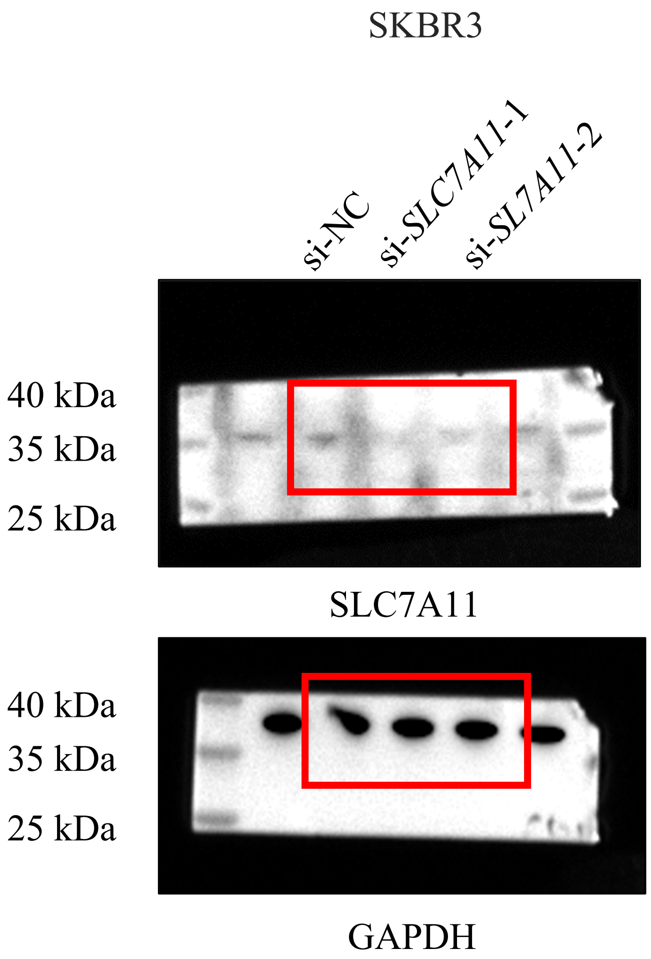
SKBR3
si-NC
si-SLC7A11-1
si-SL7A11-2
40 kDa
35 kDa
25 kDa
SLC7A11
40 kDa
35 kDa
25 kDa
GAPDH

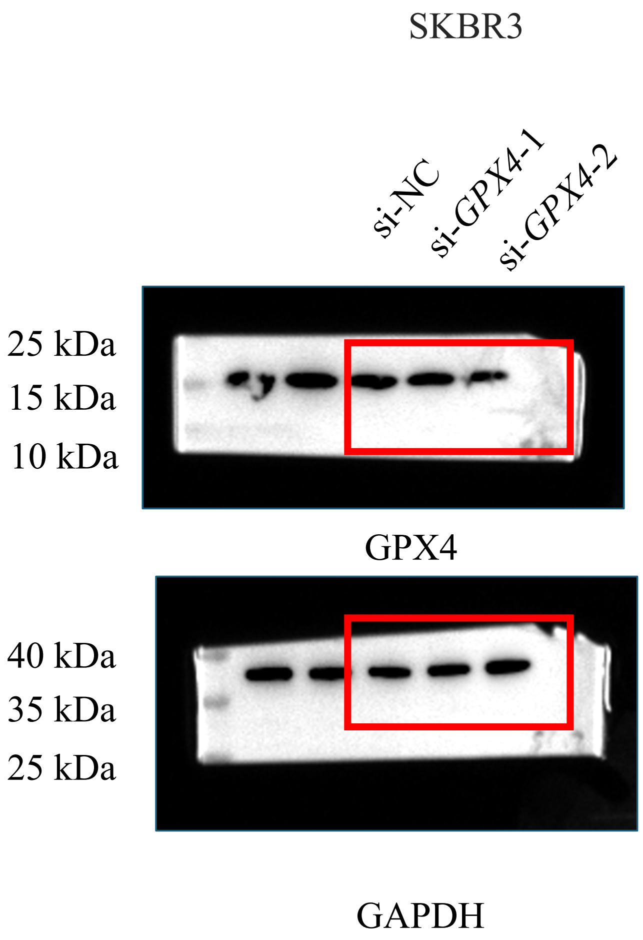
SKBR3
si-NC
si-GPX4-1
si-GPX4-2
25 kDa
15 kDa
10 kDa
GPX4
40 kDa
35 kDa
25 kDa
GAPDH
