# Supplementary material for: Targeting SLC7A11-mediated cysteine metabolism for the treatment of trastuzumab-resistant HER2-positive breast cancer
Source: eLife. 2025 Jun 4;14:RP103953. doi: 10.7554/eLife.103953 (PMC12136593; doi:10.7554/eLife.103953)
Supplement: Figure 5—source data 1. [file elife-103953-fig5-data1.zip › Figure 5-source data 1/Figure 5D,E.pdf]

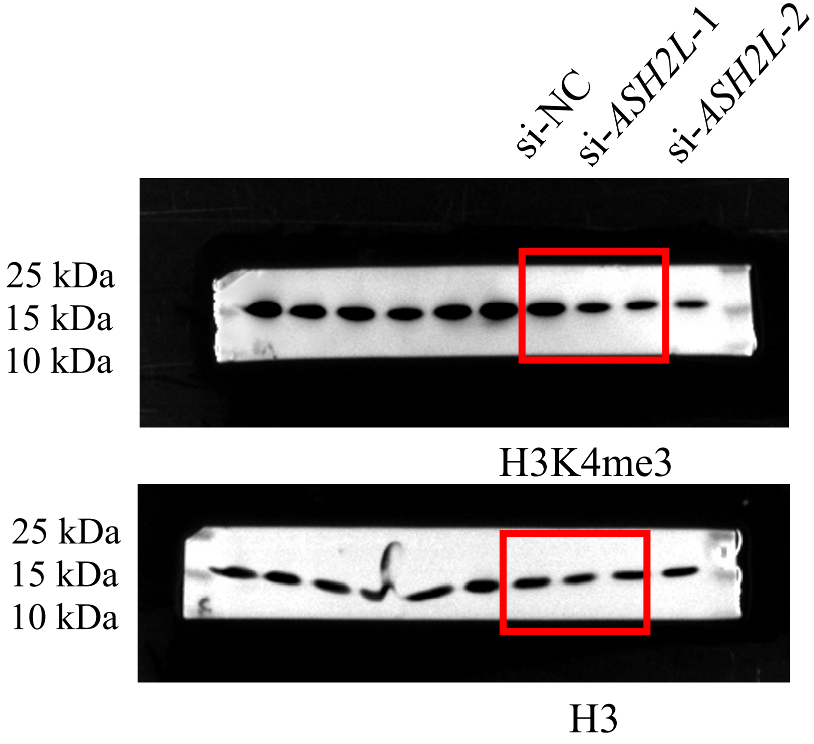
si-NC
si-ASH2L-1
si-ASH2L-2
25 kDa
15 kDa
10 kDa
H3K4me3
25 kDa
15 kDa
10 kDa
H3

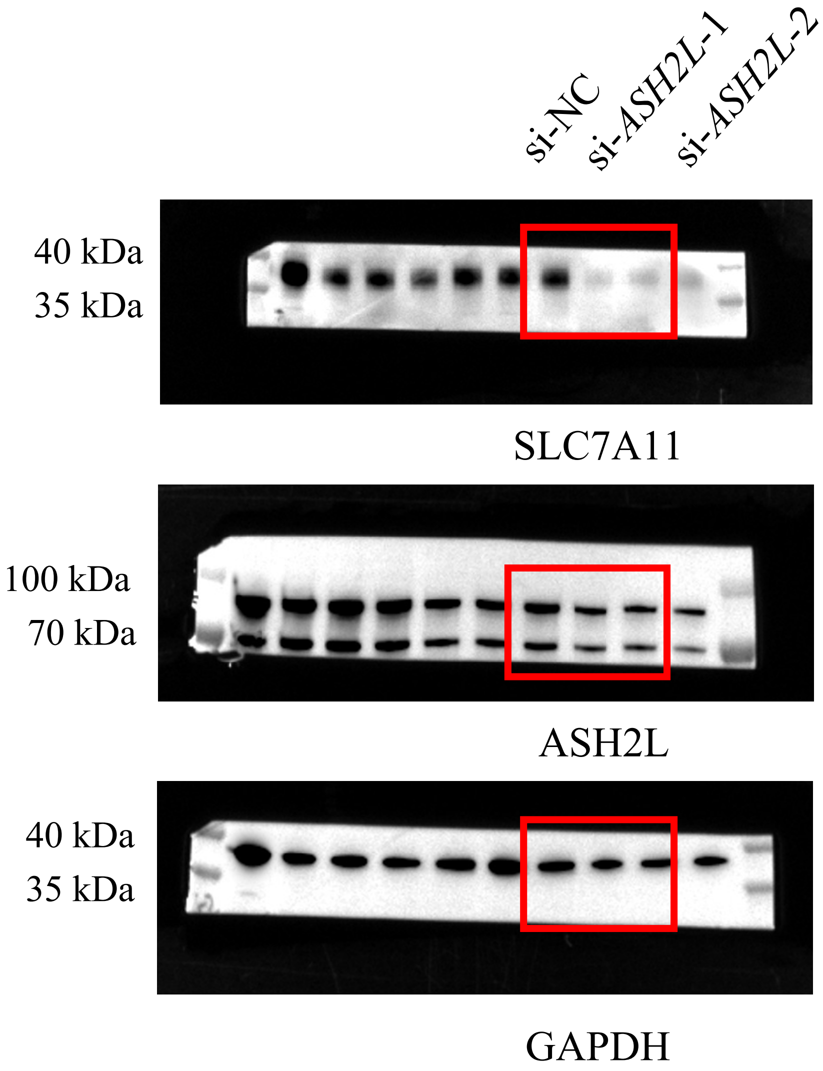
si-NC
si-ASH2L-1
si-ASH2L-2
40 kDa
35 kDa
SLC7A11
100 kDa
70 kDa
ASH2L
40 kDa
35 kDa
GAPDH
