# Supplementary material for: Targeting SLC7A11-mediated cysteine metabolism for the treatment of trastuzumab-resistant HER2-positive breast cancer
Source: eLife. 2025 Jun 4;14:RP103953. doi: 10.7554/eLife.103953 (PMC12136593; doi:10.7554/eLife.103953)
Supplement: Supplementary file 1. — Table 1. Oligonucleotide sequences of siRNAs. Table 2. Oligonucleotides sequences of small guide RNAs (sgRNAs). Table 3. PCR primers sequences for ChIP and MeDIP tests. [file elife-103953-supp1.docx]

| **siRNA** | **5'-3' sequence** | |
| --- | --- | --- |
| si-ASH2L-1 | sense strand | CGAAGACAAUGUUCUCCAA(dT)(dT) |
|  | antisense strand | UUGGAGAACAUUGUCUUCG(dT)(dT) |
| si-ASH2L-2 | sense strand | GCUGACACAUUUGGCAUAGAU |
|  | antisense strand | AUCUAUGCCAAAUGUGUCAGC |
| si-SLC7A11-1 | sense strand | GGAGUUAUGCAGCUAAUUA(dT)(dT) |
|  | antisense strand | UAAUUAGCUGCAUAACUCC(dT)(dT) |
| si-SLC7A11-2 | sense strand | CUACUUUACGACCAUUAAU(dT)(dT) |
|  | antisense strand | AUUAAUGGUCGUAAAGUAG(dT)(dT) |
| si-GPX4-1 | sense strand | GGAGUAACGAAGAGAUCAA(dT)(dT) |
|  | antisense strand | UUGAUCUCUUCGUUACUCC(dT)(dT) |
| si-GPX4-2 | sense strand | GGAAGUGGAUGAAGAUCCA(dT)(dT) |
|  | antisense strand | UGGAUCUUCAUCCACUUCC(dT)(dT) |

**Supplementary File 1-Table 1. Oligonucleotides sequences of siRNAs.**

| **sgRNA** | **5'-3' sequence** |
| --- | --- |
| SLC7A11-sgRNA-1 | (mU)*(mA)*(mC)*GAAAAAUAAGCCCAACGGUUUUAGAGCUAGAAAUAGCAAGUUAAAAUAAGGCUAGUCCGUUAUCAACUUGAAAAAGUGGCACCGAGUCGGUGCU*(mU)*(mU)*(mU) |
| SLC7A11-sgRNA-2 | (mA)*(mU)*(mG)*AAUUGAUUGGUACACAUGUUUUAGAGCUAGAAAUAGCAAGUUAAAAUAAGGCUAGUCCGUUAUCAACUUGAAAAAGUGGCACCGAGUCGGUGCU*(mU)*(mU)*(mU) |

**Supplementary File 1-Table 2. Oligonucleotides sequences of sgRNAs.**

| **PCR primers** | **5'-3' sequence** | |
| --- | --- | --- |
| SLC7A11-ChIP | Forward | CTCAGCTTCCTCATGGGCTT |
|  | Reverse | GCTGAGTAATGCTGGAGGCT |
| SLC7A11-MeDIP | Forward | CTCCTGTTCCCATGTGTACCAA |
|  | Reserve | AGTTCTCTAGATGAGTGTCAAACCA |

**Supplementary File 1-Table 3. PCR primers sequences for ChIP and MeDIP tests.**
